# Impact of deltamethrin selection on *kdr* mutations and insecticide detoxifying enzymes in *Aedes aegypti* from Mexico

**DOI:** 10.1186/s13071-020-04093-3

**Published:** 2020-05-06

**Authors:** Yamili Contreras-Perera, Gustavo Ponce-Garcia, Karina Villanueva-Segura, Beatriz Lopez-Monroy, Iram P. Rodríguez-Sanchez, Audrey Lenhart, Pablo Manrique-Saide, Adriana E. Flores

**Affiliations:** 1 Facultad de Ciencias Biologicas, Universidad Autonoma de Nuevo Leon, Cd, Universitaria, San Nicolas de los Garza, N.L. CP. 66455 Mexico; 2 Division of Parasitic Diseases and Malaria, Centers for Disease Control and Prevention, Atlanta, GA USA; 3 Unidad Colaborativa para Bioensayos Entomologicos, Universidad Autonoma de Yucatan, Campus de Ciencias Biologicas y Agropecuarias, Merida, Yucatan Mexico

**Keywords:** *Aedes aegypti*, Heritability, *kdr* mutations, V410L, V1016I, F1534C, Metabolic detoxification

## Abstract

**Background:**

Insecticide resistance is a serious problem for vector control programmes worldwide. Resistance is commonly attributed to mutations at the insecticide’s target site or increased activity of detoxification enzymes.

**Methods:**

We determined the knockdown concentration (KC_50_) and lethal concentration (LC_50_) of deltamethrin in six natural populations of adult *Aedes aegypti* from southeastern Mexico. These populations were then selected over five generations using the LC_50_ from the preceding generation that underwent selection, and the heritability of deltamethrin resistance was quantified. For each generation, we also determined the frequency of the *kdr* alleles L410, I1016 and C1534, and the levels of activity of three enzyme families (α- and β-esterases, mixed-function oxidases and glutathione S-transferases (GST)) associated with insecticide detoxification.

**Results:**

There was an increase in KC_50_ and LC_50_ values in the subsequent generations of selection with deltamethrin (F_S5_*vs* F_S0_). According to the resistance ratios (RRs), we detected increases in LC_50_ ranging from 1.5 to 5.6 times the values of the parental generation and in KC_50_ ranging from 1.3–3.8 times the values of the parental generation. Triple homozygous mutant individuals (tri-locus, LL/II/CC) were present in the parental generations and increased in frequency after selection. The frequency of L410 increased from 1.18-fold to 2.63-fold after selection with deltamethrin (F_S5_*vs* F_S0_) in the populations analyzed; for I1016 an increase between 1.19-fold to 2.79-fold was observed, and C1534 was fixed in all populations after deltamethrin selection. Enzymatic activity varied significantly over the generations of selection. However, only α- esterase activity remained elevated in multiple populations after five generations of deltamethrin selection. We observed an increase in the mean activity levels of GSTs in two of the six populations analyzed.

**Conclusions:**

The high levels of resistance and their association with high frequencies of *kdr* mutations (V410L, V1016I and F1534C) obtained through artificial selection, suggest an important role of these mutations in conferring resistance to deltamethrin. We highlight the need to implement strategies that involve the monitoring of *kdr* frequencies in insecticide resistance monitoring and management programmes.
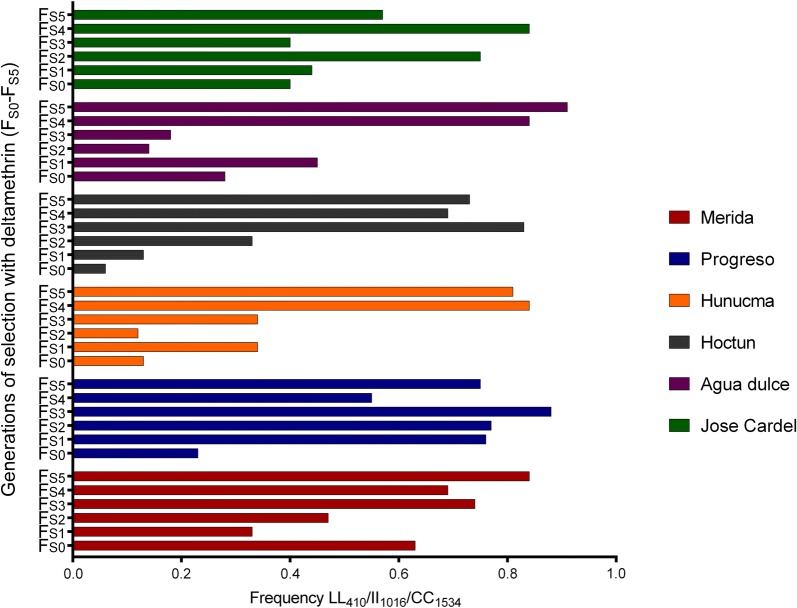

## Background

*Aedes aegypti* (L.) is an arbovirus vector of great public health importance, and is the principal vector transmitting dengue, chikungunya and Zika viruses in the Americas [1–3]. Currently, there are no widely licensed vaccines or specific treatments for any of these diseases, rendering vector control the principal strategy for preventing their transmission. The reduction or elimination of larval habitats and chemical control with the use of larvicides and adulticides are the main strategies to reduce vector-human contact and break the transmission cycles of these diseases [4]. The insecticides used for such purposes worldwide are mainly from the following chemical groups: organophosphates, organochlorines, carbamates and pyrethroids [5, 6]. Pyrethroids are often the first line of defense to break the transmission of these diseases, and they are the most commonly used worldwide due to their low toxicity to mammals including humans [7, 8].

In Mexico, the application of pyrethroid insecticides is regulated by the Ministry of Health, and their use dates back to 1999 when the use of DDT was banned in the health sector. At this time, permethrin became the insecticide of choice for space spraying and deltamethrin for indoor residual spraying [9]. In 2011, the use of permethrin was discontinued; however, deltamethrin remained on the list of insecticides approved for use as an adulticide in indoor residual spraying. In 2012 other pyrethroids were added to the list; however, deltamethrin remained, and in 2016 its use for space spraying applications was also approved, a practice that remains to date [10]. The use of pyrethroids in Mexico has been favored for more than 20 years, which led to widespread resistance in mosquito populations [11–13].

Insecticide resistance is one of the main obstacles to the success of vector control programmes [12, 14, 15]. Two of the principal mechanisms that cause resistance in mosquitoes are alterations at the target site of the insecticide, including knockdown resistance (*kdr*) mutations on the voltage-gated sodium channel gene (*vgsc*), and increased metabolic activity [16, 17]. Target-site resistance arising from *kdr* mutations can cause resistance to both DDT and pyrethroids, since both types of insecticides target sodium channels at the axon level [18].

Since the discovery of the L1014F mutation in *Musca domestica* and since the determination of its role in insecticide resistance [19], more than 50 mutations have been described for the *vgsc* gene in different pests and disease vectors [20]. Thirteen of these sequence changes are commonly found in *Ae. aegypti* in ten different positions covering five regions of the *vgsc* gene (IIS4-5, IIS5-6, IIS-6, IIIS6 and IVS5), identified at positions 410 (V → L), 419 (V → L), 923 (G → I), 982 (L → W), 989 (S → P), 1011 (I → M or V), 1016 (V → G or I), 1520 (T → I), 1534 (F → C or L), and 1763 (D → Y) [21–29]. It should be noted that only five of the mutations described for this species have been functionally associated with sensitivity to pyrethroids and DDT by heterologous expression and electrophysiology assays; only S989P, I1011M, V1016G, F1534C and V410L have been confirmed [20, 27, 30, 31]. Similarly, it is important to note that mutations are usually associated with a specific geographic sector, for example, mutation V1016G has been described in Southeast Asia but not in the Americas [32].

In Mexico, mutations F1534C, V1016I and V410L have been reported in populations of *Ae. aegypti* in different states, sites where their temporal distribution indicates the absence of mutant alleles in 2000, with a gradual increase in these mutations during the period of 2002–2008, up to fixation of the mutant alleles in 2016, showing an allelic frequency for the three simultaneous changes of 0.47 [30]. Of these mutations, the change from phenylalanine to cysteine at position 1534 was described initially by [25], who associated its occurrence with pyrethroid resistance; however, confirmation by heterologous expression and electrophysiology has demonstrated that this change reduces sensitivity of VGSC to type I but not type II pyrethroids [33]. The presence of this mutation has been linked to the development of low resistance levels and its association with other gene changes to the establishment of a more resistant phenotype. Such is the case of its association with V1016I, described by Saavedra et al. [22], where its participation in the reduction of sensitivity of VGSC has not been identified when appearing as a single mutation [31]. An analysis of the frequency of V1016I and F1534C in 24 populations of *Ae. aegypti* in Mexico showed that the most likely evolutionary process is the appearance of F1534C and then V1016I, this being corroborated by the low fitness of haplotype I1016/F1534 [34] and the increase in insecticide resistance expressed in *Xenopus* oocytes when inducing the two mutations [31]. Temporal distribution studies of this mutation in Mexico identified an increase in the frequency of the I1016 mutant allele during the period of 1996 to 2000 with a frequency of 0.04% up to an increase to 33.2% in 2007 to 2009 [35].

The V410L mutation has been recently described by Haddi et al. [27], who determined their association with resistance to type I and II pyrethroids. This mutation has been studied in Mexico in populations of *Ae. aegypti* collected between 2000 and 2016 where its appearance was reported in 2002 in heterozygous individuals collected in Coatzacoalcos, Veracruz, and where the increase in the frequency of L410 was also documented as being as high as 0.9 in populations collected in 2014 in Merida, Yucatan [30]. A similar pattern was found for 26 populations of *Ae. aegypti* collected in the eastern part of Mexico, showing a frequency of the L410 allele close to the one in populations from Minatitlan and Jose Cardel in Veracruz and Cancun in Quintana Roo (0.99, 0.97 and 0.93, respectively), but the average values, as well as the interval of frequencies observed in populations ranged from 0.3 to 0.99 [36].

Metabolic resistance to pyrethroids is mediated mainly by glutathione S-transferases (GSTs), esterases and mixed-function oxidases (MFOs) [37–39]. In Mexico, Flores et al. [14] previously reported high levels of α- and β-esterase activity related to permethrin selection in populations of *Ae. aegypti* from Baja California Norte and Sur. The increased activity of these enzymes has also been reported in populations from the state of Sonora in a study of permethrin resistance and in the state of Veracruz in a study of chlorpyrifos resistance [40, 41]. A further study reported high levels of GST activity in the state of Guerrero, which was found to be related to resistance to DDT [12].

Given the background levels of pyrethroid and DDT resistance in many parts of Mexico, the extent to which ongoing insecticide pressure selects for specific mechanisms of resistance is not well understood. To better understand how insecticide exposure selects for resistance we evaluate the effect of laboratory selection with deltamethrin on the underlying resistance mechanisms in populations of *Ae. aegypti* from southeastern Mexico, over five generations of selection.

## Methods

### Biological material

**Fig. 1:**
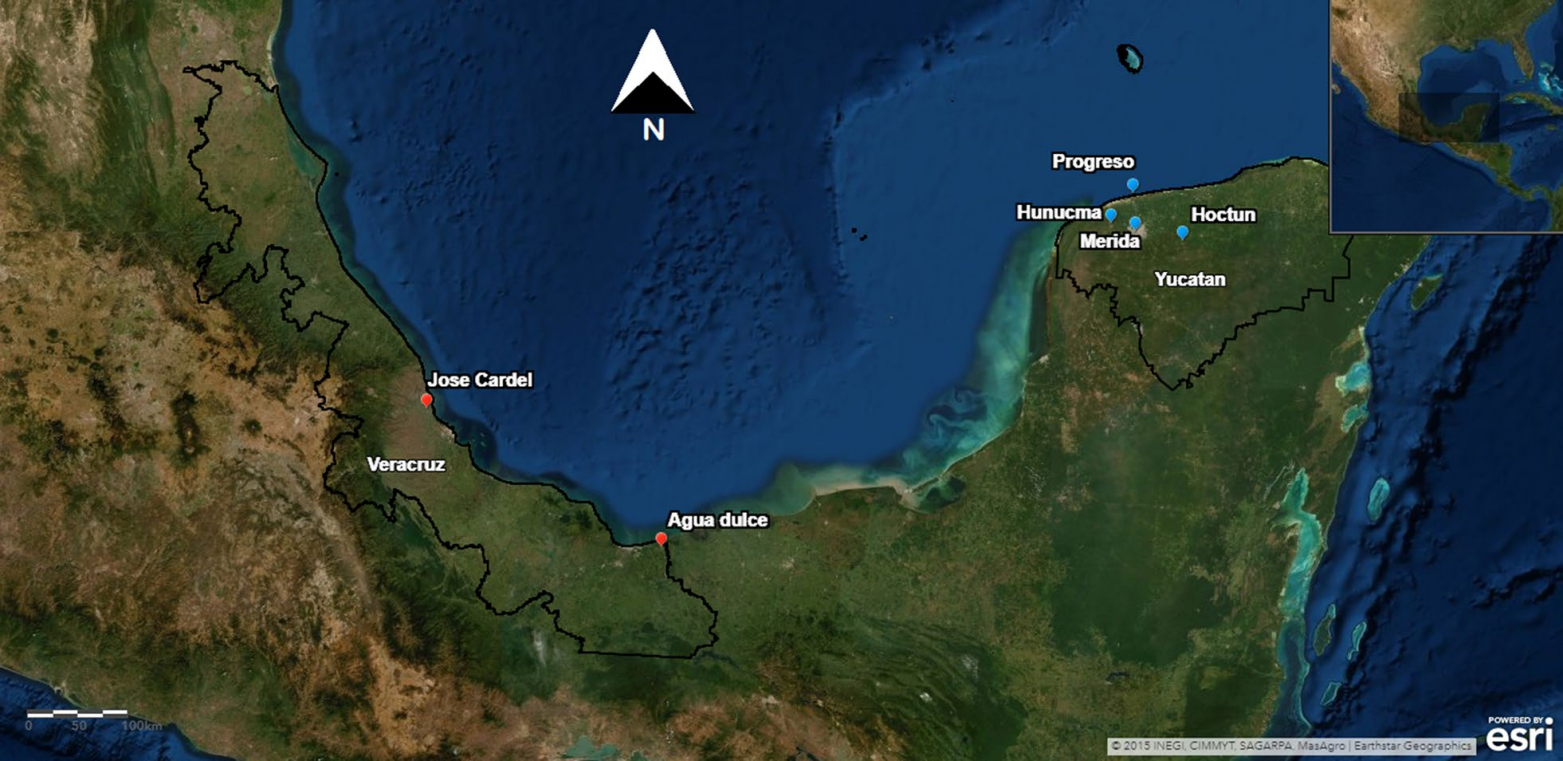
Collection sites of *Aedes aegypti* from Veracruz and Yucatan states from Mexico

Eggs and larvae of *Ae. aegypti* were collected during 2014 from six sites: Merida, Progreso, Hunucma, and Hoctun in the State of Yucatan; and Agua Dulce and Jose Cardel, in the State of Veracruz (Table 1, Fig. 1). The criteria for site collection were: high incidence of dengue and frequent application of insecticides for its control; high infestation index of homes with *Ae. aegypti*; and previous registration of insecticide resistance. The collection of the entomological material was carried out in peridomestic sites. In each, entomological inspections were performed for the collection of stages of *Ae. aegypti* in at least 20 potential breeding places per site, such as containers, tires and flowerpots.

**Table 1:** Collection sites, geographical coordinates

State	Municipality	Location	Coordinates
Yucatan	Merida	Manzana 115	20° 56′ 42″ N, 89° 38′ 36″ W
	Progreso		21° 16′ 52″ N, 89° 39′ 54″ W
	Hunucma		21° 00′ 58″ N, 89° 52′ 38″ W
	Hoctun		20° 51′ 50″ N, 89° 12′ 03″ W
Veracruz	Agua dulce	Agua dulce	18° 08′ 33″ N, 94° 08′ 36″ W
	La Antigua	Jose Cardel	19° 22′ 15″ N, 96° 22′ 35″ W

The biological material obtained from the field was reared under insectary conditions at 26 ± 2 °C and 70–80% relative humidity with a 12h:12h (light:dark) photoperiod. The adults obtained from field-collected material were allowed to intermate, and the eggs resulting were designated F_S0_ (without previous selection). The New Orleans strain (NO) was used as a susceptible reference in the study, this strain was originally obtained from the CDC (Atlanta, GA, USA) and has been maintained since 2002.

### Bioassays

The bioassays consisted of exposing 20–25 non-blood-fed, 2–3-day-old F_S0_ female mosquitoes to different concentrations of the pyrethroid deltamethrin (> 98% purity; Chem Service, West Chester, PA, USA) based on the bottle bioassay methodology described by the CDC [42]. Each bioassay consisted of bottles containing different concentrations of deltamethrin resulting in mortalities between 9–90%, with at least three replicates for each concentration, and an untreated control bottle. The numbers of knocked-down mosquitoes were recorded at 1 h. After 1 h of exposure, all mosquitoes were gently transferred to a recovery container without insecticide and were offered a cotton ball soaked in a sugar solution. Mortality was recorded at 24 h. Both the bottles and recovery containers were kept at 24 ± 2 °C and 70% RH.

Rates of *kdr* and 24 h recovery were analyzed using a logistic regression model, QCal (https://sourceforge.net/projects/irmaproj/files/Qcal/) [43]. We determined the KC_50_ (concentration causing 50% knockdown) after 1 h of exposure. The LC_50_ (concentration causing 50% mortality) was estimated from mortality data 24 h after recovery. The confidence intervals were calculated using a significance level of *α *= 0.05. The mortalities were corrected according to Abbott’s formula [44] when mortality was observed in the control bottles. The resistance ratios (RR) were calculated by dividing the KC_50_ or LC_50_ by the KC_50_ or LC_50_ of the susceptible New Orleans (NO) *Ae. aegypti* reference strain. The magnitude of resistance was classified as high (RR > 10-fold), moderate (RR between 5–10-fold) or low (RR < 5-fold) according to the criteria proposed by Mazarri & Georghiou [45].

### Selection with deltamethrin

Selection cohorts were generated according to the methodology described by Saavedra et al. [46]. The selection was carried out exposing 650–1000 F_S1_ males and females (1:4) for 1 h to the LC_50_ obtained for the previous generation (F_S0_) by the method described above. The survivors at 24 h were transferred to mosquito cages for breeding the subsequent generation (F_S2_). The dose response parameters were calculated again for the F_S1_ cohort and were used to select the next generation (F_S2_). This procedure was repeated for each generation of selection until F_S5_. The New Orleans strain was tested at the same time as the F_S0_ and was not tested in each generation of selection.

KC_50_ and LC_50_ values were compared between generations to monitor changes in resistance. The KC_50_ and LC_50_ values were considered significantly different if their 95% confidence intervals did not overlap.

### Realized heritability

Resistance risk assessment was made by calculating realized heritability values (h^2^) in all selection cohorts for the knockdown (KC_50_) and lethal (LC_50_) parameters as described by Tabashnik [47]. The heritability index (h^2^= R/S) is calculated as the ratio of the response to selection (R) to the selection differential (S) according to the artificial selection technique of Falconer [48] and is related to the additive genetic variance for a trait. A low h^2^ predicts no additive genetic variance for a trait and a poor or very slow response to artificial selection, while a high h^2^ predicts a large additive genetic variance at one or a few loci that govern a trait and predicts a rapid response to artificial selection.

The selection differential is expressed as the product of selection intensity (i) and phenotypic standard deviation (S= iσ). The response to selection was determined for each generation as the difference in population means between subsequent generations with the probit analysis: R = log (Final KC_50_ or LC_50_) − log (Initial KC_50_ or LC_50_)/n, where final KC_50_ or LC_50_ area the values of the offspring after n generations of selection and initial KC_50_ or LC_50_ are the values of the parental generation before n generations of selection. The selection intensity was calculated from the proportion of surviving individuals in the examined population. The difference between KC_50_ or LC_50_ was calculated on a logarithmic scale because the logarithm of tolerance was assumed to be normally distributed. The phenotypic standard deviation at t-th generation (σ_t_) was obtained as the inverse of the regression slope: σ_t_= 1/b_t_. The parameters (*R*, *i* and σ) were determined at every generation and the realized heritability was estimated as the regression coefficient of cumulative responses on cumulative selection differentials [48].

### Molecular assays

DNA was extracted from individual mosquitoes by the technique described by Coen et al. [49], and the DNA pellet was resuspended in 30 μl of ultrapure molecular grade water (Corning Cellgro^TM^, Manassas, VA, USA). The quantity and quality of DNA were determined using a NanoDrop spectrophotometer 2000 (Thermo Fisher Scientific, Woonsocket, RI, USA).

The *kdr* alleles 1534C, 1016I and 410L were detected by endpoint PCR using a Bio-Rad (Hercules, CA, USA) T100TM thermocycler using the primers for each of the mutations described by Saavedra et al. [22, 30] and Yanola et al. [25].

The primers used to detect the V1016I mutation were: V1016fw (5′-GCG GGC AGG GCG GCG GGG GCG GGG CCA CAA ATT GTT TCC CAC CCG CAC CGG-3′); I1016fw (5′-GCG GGC ACA AAT TGT TTC CCA CCC GCA CTG A-3′); and I1016R (5′-TGA TGA ACC SGA ATT GGA CAA AAG C-3′). The PCR was carried out in a reaction mixture of 12.5 µl containing 1.25 µl of 10× buffer (Invitrogen, Carlsbad, CA, USA), 1.5 mM MgCl_2_, 0.2 mM dNTPs, 5 µM of each primer, 100 ng genomic DNA, and 2 U Taq polymerase (Invitrogen). The PCR reaction conditions were: 95 °C for 5 min; 29 cycles of 95 °C for 1 min, 60 °C for 1 min, 72 °C for 1 min 15 s; and a final extension step at 72 °C for 10 min [22].

The primers used to detect the F1534C mutation were: C1534fw (5′-GCG GGC AGG GCG GCG GGG GCG GGG CCT CTA CTT TGT GTT CTT CAT CAT GTG-3′); F1534fw (5′-GCG GGC TCT ACT TTG TGT TCT TCA TCA TAT T-3′); and F1534R (5′-TCT GCT CGT TGA AGT TGT CGA T-3′). The PCR was carried out in a reaction mixture of 12.5 µl containing 1.25 µl of 10× buffer (Invitrogen), 1.5 mM MgCl_2_, 0.1 mM dNTPs, 0.5 µM of each primer, 100 ng genomic DNA, and 1 U Taq polymerase (Invitrogen). The PCR reaction conditions were: 95 °C for 4 min; 35 cycles of 95 °C for 1 min, 57 °C for 1 min, 72 °C for 1 min; and a final extension step at 72 °C for 4 min [50].

The primers used to detect the V410L mutation were: V410fw (5′-GCG GGC AGG GCG GCG GGG GCG GGG CCA TCT TCT TGG GTT CGT TCT ACC GTG-3′), L410fw (5′-GCG GGC ATC TTC TTG GGT TCG TTC TAC CAT T-3′); and 410R (5′-TTC TTC CTC GGC GGC CTC TT-3′). Amplification was performed following the methodology described by Villanueva-Segura et al. [36]. In a 1.5 ml tube, a master mix was prepared as follows: 12.50 μl of GoTaq (Promega, Madison, WI, USA) was mixed with 11.35 μl of nuclease-free H_2_O (NFW, Promega), and 1 μM of each of the primers (V410fw, L410fw and 410rev) was then added. The master mix (24 μl) was then placed in 0.2 ml stoppered tubes followed by 1 μl of mosquito genomic DNA (~25 ng). The tubes were centrifuged for 1 min at 3300× *rpm*, then placed in a T100 thermocycler (Bio-Rad) with the following temperature program: 3 min at 95 °C; 30 cycles of 1 min at 95 °C, 20 s at 56 °C and 20 s at 72 °C; followed by 5 min at 72 °C.

DNA from the New Orleans susceptible strain was used as a negative control for the *kdr* assays, and previously genotyped individuals were used as positive controls.

The PCR products for the V410L, V1016I and F1534C assays were visualized on 2.5%, 3% and 4% agarose gels, respectively, using an UVITEC (Cambridge, UK) imaging system.

The frequencies of the alleles were determined for each population at each generation. We verified that the populations at the parental generation (F_S0_) were in Hardy-Weinberg equilibrium by means of Chi-square analysis. In addition, Wright’s F_IS_ inbreeding coefficient was estimated, along with Wald’s correction [51, 52].

### Biochemical assays

Thirty-two 2–4-day-old unfed female mosquitoes were individually homogenized in 2 ml of 0.01M phosphate buffer (pH 7.2). Aliquots of 100 μl of the homogenate were distributed in triplicate in flat-bottom microplates (Corning, Tewksbury, MA, USA) for testing the activity levels of the following families of enzymes: α- and β-esterases, MFOs, and GSTs, based on mosquito-specific biochemical assay protocols [53–56].

### α- and β-esterases

To measure the activity levels of α- and β-esterases, 100 μl of either α- or β-naphthyl acetate (Sigma-Aldrich, St. Louis, MO,USA) dissolved in acetone (CTR Scientific, Monterrey, N.L., Mexico), and phosphate buffer (KPO_4_, pH 7.2) was added to each well and incubated for 20 min at room temperature. Subsequently, 100 μl of fast blue (tetrazotized O-dianisidine (Sigma-Aldrich) dissolved in distilled water) was added to each well and the microplates incubated for another 4 min, before absorbance was read at 540 nm using a spectrophotometer (ASYS Hitech GmbH, Eugendorf, Austria).

### MFOs

200 μl of TMBZ (3,3,5,5-tetramethyl-benzidine dihydrochloride (Sigma-Aldrich) previously dissolved in methanol (Jalmek, Monterrey, N.L. Mexico) and 0.25 M sodium acetate buffer pH 5.0) were added to each well. Subsequently 25 μl of 3% hydrogen peroxide (H_2_O_2_) (Jalmek) was added to each well. After 10 min of incubation at room temperature the microplate was read at a wavelength of 620 nm.

### GSTs

100 μl of reduced glutathione (GSH; Sigma-Aldrich) and 100 μl 1-chloro-2,4 dinitrobenzene (CDNB; Sigma-Aldrich) previously diluted in acetone and KPO_4_ buffer were added to each well. Absorbance was read at 340 nm immediately (T0) and after 10 min of incubation (T10). The values obtained after subtracting the initial reading (T0) from the reading at 10 min (T10) were used for the statistical analyses.

Total protein content was determined by the Bradford method [56], which was used to correct the activity values for all of the enzymes evaluated [57].

Positive and negative controls were included for MFOs and esterases. The same volume of homogenate which was used in the respective assays was used in running the controls. For the α- and β-esterases, α- and β -naphthyl acetate solution was used as positive controls, respectively. Cytochrome-C (Merck, Darmstadt, Germany) solution was the positive control for the MFO assay. KPO_4_ buffer was used as a negative control for each biochemical assay.

### Calibration curves

The calibration curves were generated using control solutions for each enzyme family. α- or β-naphthol (Sigma-Aldrich) was used for α-esterases and β-esterases, with a concentration range of 0.3 to 5 μg/μl and 0.5 to 4 μg/μl, respectively. Cytochrome C concentrations from 0.002 to 0.65 μg/μl were used for MFOs, and 0.1 to 6.5 μg/μl BSA solutions were used for total protein determination.

The absorbance values obtained for each enzyme were used to calculate the mean absorbance values per mosquito, which was then converted to enzyme activity after accounting for the homogenization volume, total protein content and the activity unit considered for each enzyme. Each value was multiplied by a conversion factor that was obtained from the calibration curves [57, 58].

The mean activity values underwent an analysis of variance (ANOVA, *P* < 0.05) and Tukey’s multiple comparison of means (*P* < 0.05). Normality of the variance was verified by the Bartlett test. Statistical analyses were carried out with GraphPad Prism v.7 (GraphPad Software, Inc, Version 6.01, La Jolla, CA, USA; https://www.graphpad.com). Activity levels for all enzymes were determined in the parental population and in each deltamethrin-selected generation (F_S0_–F_S5_).

The activity values corresponding to the 99th percentile of the New Orleans reference strain were calculated for each enzyme family. The average enzymatic activity was classified as non-altered (NA) when < 15% of individuals did not exceed the 99th percentile of the reference strain, incipiently altered (IA) if 15–50% of the individuals exceeded the 99th percentile, and altered (A) when this percentage exceeded 50%, according to the criteria established by Montella et al. [59]. Additionally, the LC_50_ values and mean enzyme activity values of the parental generation and each selected generation (F_S1_–F_S5_) underwent linear regression analysis. Correlation coefficients were calculated to determine the degree of association between the two variables.

## Results

### Bioassays

**Fig. 2:**
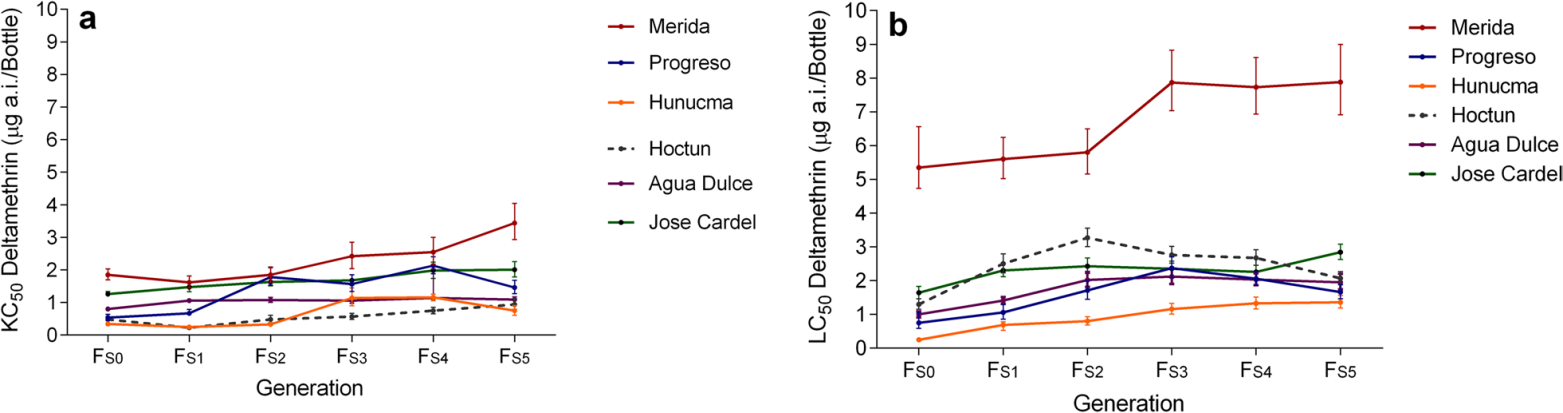
**a** Knockdown concentrations values (KC_50_) as response to the selection with deltamethrin. **b** Lethal concentrations values (LC_50_) as response to the selection with deltamethrin in six populations of *Aedes aegypti* through five generations

Deltamethrin KC_50_ and LC_50_ were determined for the parental generation and in each selected generation (F_S1_-F_S5_) as well as for the insecticide-susceptible New Orleans (NO) reference strain. Although some variation was observed, KC_50_ and LC_50_ generally increased with respect to the parental generation (F_S5_*vs* F_S0_) (Tables 2, 3, Fig. 2).

**Table 2:** Deltamethrin knockdown concentrations (KC_50_ and KC_90_) and resistance ratios (RR) for *Aedes aegypti* females in the parental generation (F_S0_) and the deltamethrin-selected generations (F_S1_–F_S5_)

Population	Generation	n^a^	KC^b^ μg/B				RR^c^	
			KC_50_ (CI)	KC_90_ (CI)	b^d^ ± SE	*P-*value	KC_50_	KC_90_
Merida	F_S0_	345	1.85 (1.69–2.03)	3.89 (3.27–4.63)	2.95 (0.35)	< 0.001	20	8
	F_S1_	403	1.62 (1.45–1.82)	4.65 (3.62–5.97)	2.09 (0.21)	< 0.001	18	10
	F_S2_	525	1.85 (1.63–2.09)	6.75 (4.88–9.33)	1.69 (0.19)	< 0.001	20	14
	F_S3_	540	2.42 (2.04–2.85)	15.19 (14.58–21.80)	1.19 (0.10)	< 0.001	27	33
	F_S4_	440	2.55 (2.17–3.00)	12.79 (9.01–18.16)	1.36 (0.13)	< 0.001	28	27
	F_S5_	400	3.44 (2.93–4.04)	14.84 (10.88–20.23)	1.50 (0.15)	< 0.001	38	32
Progreso	F_S0_	490	0.54 (0.45–0.64)	3.22 (2.26–4.60)	1.23 (0.11)	< 0.001	6	7
	F_S1_	526	0.67 (0.60–0.79)	3.00 (2.25–3.99)	1.50 (0.13)	< 0.001	7	7
	F_S2_	417	1.78 (1.53–2.07)	7.13 (4.84–10.50)	1.58 (0.18)	< 0.001	20	15
	F_S3_	395	1.57 (1.34–1.85)	7.04 (4.93–10.03)	1.47 (0.15)	< 0.001	17	15
	F_S4_	439	2.13 (1.88–2.40)	7.36 (5.47– 9.91)	1.77 (0.19)	< 0.001	23	16
	F_S5_	320	1.46 (1.27–1.68)	5.63 (4.57–6.93)	2.02 (0.22)	< 0.001	23	13
Hunucma	F_S0_	355	0.34 (0.28–0.40)	1.42 (1.03–1.96)	1.16 (0.09)	< 0.001	3	3
	F_S1_	460	0.25 (0.21–0.29)	1.25 (0.88–1.76)	1.37 (0.12)	< 0.001	3	3
	F_S2_	597	0.33 (0.27–0.40)	3.27 (2.13–5.01)	0.96 (0.08)	< 0.001	4	7
	F_S3_	353	1.14 (0.90–1.45)	10.19 (5.53–18.80)	1.00 (0.12)	< 0.001	13	22
	F_S4_	220	1.16 (1.06–1.26)	1.93 (1.69–2.20)	4.30 (0.62)	< 0.001	13	4
	F_S5_	180	0.75 (0.61–0.91)	2.26 (1.62–3.16)	1.98 (0.33)	< 0.001	8	5
Hoctun	F_S0_	535	0.48 (0.41–0.56)	1.85 (1.48–2.32)	1.63 (0.14)	< 0.001	5	4
	F_S1_	535	0.23 (0.19–0.27)	1.32 (0.97–1.80)	1.35 (0.10)	< 0.001	2	3
	F_S2_	470	0.48 (0.38–0.61)	5.73 (3.47–9.46)	0.88 (0.08)	< 0.001	5	12
	F_S3_	508	0.57 (0.48–0.67)	2.92 (2.13–3.99)	1.34 (0.12)	< 0.001	6	6
	F_S4_	380	0.75 (0.65–0.86)	2.61 (1.92–3.56)	1.76 (0.23)	< 0.001	8	6
	F_S5_	440	0.94 (0.79–1.12)	4.19 (3.09–5.66)	1.47 (0.14)	< 0.001	10	9
Agua Dulce	F_S0_	536	0.80 (0.76–0.84)	1.44 (1.30–1.60)	3.73 (0.30)	< 0.001	9	3
	F_S1_	480	1.06 (1.01–1.12)	1.80 (1.62–2.01)	4.15 (0.38)	< 0.001	12	4
	F_S2_	333	1.08 (1.00–1.16)	1.99 (1.70–2.33)	3.58 (0.45)	< 0.001	12	4
	F_S3_	313	1.06 (0.97–1.15)	2.06 (1.74–2.44)	3.32 (0.38)	< 0.001	12	4
	F_S4_	397	1.14 (1.05–2.03)	2.42 (2.03–2.89)	2.92 (0.32)	< 0.001	13	5
	F_S5_	440	1.09 (1.02–1.17)	2.04 (1.79–2.33)	3.53 (0.34)	< 0.001	12	5
Jose Cardel	F_S0_	420	1.26 (1.21–1.34)	2.18 (1.95–2.48)	4.01 (1.18)	< 0.001	14	5
	F_S1_	429	1.47 (1.33–1.64)	4.10 (3.28–5.12)	2.15 (0.20)	< 0.001	16	9
	F_S2_	499	1.63 (1.51–1.75)	3.73 (3.08–4.50)	2.65 (0.28)	< 0.001	18	8
	F_S3_	400	1.68 (1.53–1.85)	4.07 (3.35–4.95)	2.48 (0.24)	< 0.001	19	9
	F_S4_	400	1.98 (1.74–2.24)	6.68 (5.03–8.86)	1.80 (0.19)	< 0.001	22	14
	F_S5_	360	2.01 (1.79–2.26)	5.68 (4.35–7.42)	2.12 (0.23)	< 0.001	22	12
New Orleans (NO)^e^	–	492	0.09 (0.08–0.11)	0.46 (0.32-0.65)	1.43 (0.13)	< 0.001	–	–

^a^ sample size

^b^ KC: 50% and 90% knockdown concentrations in micrograms per bottle, 95% confidence intervals

^c^ RR: resistance ratio KC_50_ field strain/ KC_50_ susceptible strain

^d^ b: logistic regression slope (standard error)

^e^ New Orleans: susceptible reference strain

When comparing the LC_50_ values obtained from the parental generations to the NO strain, five of the populations (Merida, Progreso, Hoctun, Agua Dulce and Jose Cardel) were highly resistant to deltamethrin with an RR_LC50_ of 18–134-fold, and only one population (Hunucma) showed moderate resistance (RR_LC50_ of 6-fold) (Table 3).

**Table 3:** Deltamethrin knockdown concentrations (LC_50_ and LC_90_) and resistance ratios (RR) for *Aedes aegypti* females in the parental generation (F_S0_) and the deltamethrin-selected generations (F_S1_–F_S5_)

Population	Generation	n^a^	LC^b^ μg/B				RR^c^	
			LC_50_ (CI)	LC_90_ (CI)	b^d^ ± SE	*P-*value	LC_50_	LC_90_
Merida	F_S0_	466	5.35 (4.73–6.56)	42.11 (27.77–63.84)	1.06 (0.09)	< 0.001	134	140
	F_S1_	584	5.60 (5.02–6.24)	18.63 (15.07–23.03)	1.82 (0.15)	< 0.001	140	62
	F_S2_	525	5.80 (5.16–6.49)	19.32 (19.96–30.27)	1.82 (0.18)	< 0.001	145	64
	F_S3_	520	7.87 (7.03–8.82)	24.58 (19.96–30.27)	1.93 (0.17)	< 0.001	196	82
	F_S4_	501	7.73 (6.93–8.61)	24.15 (19.69–29.62)	1.92 (0.17)	< 0.001	193	80
	F_S5_	389	7.88 (6.91–9.00)	26.31 (19.84–34.89)	1.82 (0.21)	< 0.001	197	88
Progreso	F_S0_	650	0.75 (0.59–1.06)	7.06 (4.83–10.31)	0.97 (0.08)	< 0.001	18	23
	F_S1_	473	1.06 (0.86–1.29)	7.72 (5.21–11.44)	1.10 (0.09)	< 0.001	26	26
	F_S2_	415	1.71 (1.45–2.01)	7.19 (5.30–9.76)	1.53 (0.15)	< 0.001	43	24
	F_S3_	458	2.37 (2.08–2.71)	8.97 (6.74–11.95)	1.65 (0.15)	< 0.001	59	30
	F_S4_	461	2.06 (1.86–2.28)	5.63 (4.57–6.93)	2.18 (0.20)	< 0.001	51	19
	F_S5_	320	1.66 (1.46–1.88)	4.32 (3.23–5.78)	2.02 (0.22)	< 0.001	36	14
Hunucma	F_S0_	424	0.25 (0.20–0.29)	1.43 (0.99–2.05)	1.24 (0.12)	< 0.001	6	5
	F_S1_	359	0.69 (0.53–0.78)	3.50 (2.32–5.28)	1.30 (0.14)	< 0.001	17	12
	F_S2_	497	0.80 (0.69–0.93)	3.63 (2.68–4.91)	1.45 (0.14)	< 0.001	20	12
	F_S3_	299	1.16 (1.01–1.33)	3.61 (2.69–4.83)	1.93 (0.23)	< 0.001	29	12
	F_S4_	260	1.33 (1.16–1.52)	3.26 (2.62–4.04)	2.45 (0.26)	< 0.001	33	11
	F_S5_	318	1.36 (1.19–1.56)	4.29 (3.20–5.75)	1.92 (0.22)	< 0.001	34	14
Hoctun	F_S0_	532	1.30 (1.15–1.46)	4.06 (3.25–5.08)	1.92 (0.16)	< 0.001	32	13
	F_S1_	416	2.50 (2.23–2.79)	6.67 (5.51–8.07)	2.23 (0.22)	< 0.001	62	22
	F_S2_	467	3.27 (3.01–3.55)	7.43 (6.28–8.78)	2.67 (0.27)	< 0.001	82	23
	F_S3_	417	2.76 (2.52–3.02)	6.60 (5.45–8.00)	2.52 (0.26)	< 0.001	69	22
	F_S4_	457	2.67 (2.45–2.92)	6.51 (5.36–7.89)	2.47 (0.26)	< 0.001	67	22
	F_S5_	359	2.07 (1.89–2.27)	4.37 (3.72–5.13)	2.94 (0.33)	< 0.001	52	15
Agua Dulce	F_S0_	475	1.00 (0.91–1.09)	2.62 (2.14–3.21)	2.27 (0.21)	< 0.001	25	7
	F_S1_	540	1.41 (1.31–1.52)	3.15 (2.65–3.75)	2.74 (0.24)	< 0.001	32	10
	F_S2_	420	2.02 (1.81–2.25)	5.51 (4.49–6.78)	2.18 (0.21)	< 0.001	50	18
	F_S3_	457	2.12 (1.90–2.35)	6.19 (4.91–7.73)	2.05 (0.19)	< 0.001	53	20
	F_S4_	519	2.03 (1.86–2.22)	5.32 (4.40–6.44)	2.28 (0.20)	< 0.001	51	18
	F_S5_	340	1.95 (1.74–2.20)	6.40 (4.79–8.55)	1.85 (0.19)	< 0.001	49	21
Jose Cardel	F_S0_	481	1.64 (1.46–1.83)	5.22 (4.10–6.64)	1.89 (0.18)	< 0.001	41	6
	F_S1_	531	2.30 (2.12–2.49)	5.33 (4.53–6.28)	2.61 (0.25)	< 0.001	57	18
	F_S2_	460	2.43 (2.21–2.67)	6.01 (4.95–7.30)	2.42 (0.22)	< 0.001	60	20
	F_S3_	360	2.35 (2.14–2.58)	5.12 (4.33–6.06)	2.82 (0.27)	< 0.001	56	17
	F_S4_	420	2.26 (2.07–2.45)	4.93 (4.27–5.69)	2.81 (0.25)	< 0.001	56	16
	F_S5_	320	2.84 (2.63–3.08)	5.49 (4.67–6.44)	3.34 (0.34)	< 0.001	71	18
New Orleans (NO)^e^	–	492	0.04 (0.03–0.05)	0.30 (0.21–0.45)	1.11 (0.10)	< 0.001	–	–

^a^ sample size

^b^ LC: 50% and 90% lethal concentrations in micrograms per bottle, 95% confidence intervals

^c^ RR: resistance ratio LC_50_ field strain/ LC_50_ susceptible strain

^d^*b*: slope of the regression line log-Probit (standard error)

^e^ New Orleans: susceptible reference strain

With respect to knockdown in the parental generation, RR_KC50_ was high in two populations, Merida with RR_KC50_ of 20-fold and Jose Cardel with RR_KC50_ of 14-fold. Three populations (Hoctun, Progreso and Agua Dulce) showed moderate resistance with RR_KC50_ ranging between 5–9-fold, and one population (Hunucma) had a low RR_KC50_ of 3-fold (Table 2).

When the populations were selected with deltamethrin, the increase in LC_50_ was not significant for F_S1_ and F_S2_ for the Merida population (based on overlapping 95% CIs). However, there was a significant increase in LC_50_ from F_S1_ to F_S5_ in all other populations of *Ae. aegypti* (Table 3). For KC_50_, the increases were significant between all generations of selection (F_S1_-F_S5_) for the Progreso, Agua Dulce and Jose Cardel populations but were not significant for the Merida and Hunucma populations for F_S1_-F_S2_ and the Hoctun population for F_S1_-F_S3_ (Table 2).

When comparing the LC_50_ and KC_50_ values for the last deltamethrin-selected generation (F_S5_) *vs* the parental generation (F_S0_), we observed an increase of ~1.5–5.5 times for LC_50_ and ~1.4–2.7 times for KC_50_ in all populations (Fig. 2).

The results revealed that deltamethrin resistance increased in response to selection pressure (Tables 2, 3, Fig. 2). The knockdown resistance ratio (RR_KC50_) value increased from 20-fold to 38-fold from F_S0_-F_S5_ in the Merida population, from 6-fold to 23-fold for Progreso, from 3-fold to 8-fold for Hunucma, from 5-fold to 10-fold in Hoctun, and slight increases were observed in the Agua Dulce and Jose Cardel populations with 9–12-fold and 14–22-fold differences, respectively. Lethal concentrations also increased between F_S0_ and F_S5_, with the RR_LC50_ increasing in the Hunucma populations from 6-fold to 34-fold, doubling in the Agua Dulce and Progreso populations (25–49-fold and 18–36-fold, respectively) and with lower increases detected in the Merida, Hoctun and Jose Cardel populations (134–197-fold, 32–52-fold and 41–71-fold, respectively). When analyzing the relative increases in RR_KC50_ and RR_LC50_ during the selection, the Hunucma population showed the highest single-generation increase (> 0.3-fold) from F_S1_*vs* F_S2_ for KC_50_; Hunucma showed also the highest increase in RR_LC50_ (2.83) in the first selected generation relative to the parental generation (F_S1_*vs* F_S0_).

### Realized heritability

The heritability of resistance (h^2^) estimated over the generations of deltamethrin selection was highest in the Hoctun and Merida populations, with values of 0.948 and 0.900, respectively, for LC_50_. In the case of KC_50_, the highest values were obtained for the Progreso population (0.779) and Merida populations (0.519). The response to the selection (R) was also high for Merida and Progreso, for both KC_50_ and LC_50_. The selection differential (S) was high in the Progreso population for LC_50_, but in the case of KC_50_, the highest values were obtained for Hoctun and Hunucma. The number of generations required for a 10-fold increase in LC_50_, (G), is the reciprocal of the response to selection (R) [47]. Thus, for the Progreso, Merida and Hoctun populations, an estimated mean of ~ 2 generations was necessary for a 10-fold increase in LC_50_; however, for Hunucma, Agua Dulce and Jose Cardel, a mean of > 4 generations was necessary. Regarding KC_50_, an estimated mean of ~ 2 generations was necessary for a 10-fold increase for the Merida and Progreso populations, but for Hunucma, Hoctun, Jose Cardel and Agua Dulce, means of 3, ~ 6, 7 and ~ 25 generations, respectively, were necessary for a 10-fold increase (Tables 4, 5).

**Table 4:** Estimation of heritability (*h*^2^) of resistance in *Aedes aegypti* after deltamethrin selection for knockdown concentration (KC_50_)

Population	Generation	KC_50_	b	δt	Pt	it	S	ΣS	R	ΣR	*h2*
Merida	F_S0_	1.855	2.959	0.338	–	–	–	–	–	–	
	F_S1_	1.628	2.094	0.478	21.920	1.346	0.643	0.643		0.000	
	F_S2_	1.852	1.699	0.589	24.010	1.295	0.762	1.405	0.224	0.224	
	F_S3_	2.421	1.196	0.836	20.670	1.372	1.147	2.552	0.569	0.793	
	F_S4_	2.556	1.364	0.733	24.030	1.295	0.949	3.502	0.135	0.928	
	F_S5_	3.446	1.505	0.664	–	–			0.890	1.818	0.519
Progreso	F_S0_	0.541	1.231	0.812	–	–	–	–	–	–	
	F_S1_	0.673	1.500	0.667	24.770	1.271	0.847	0.847		0.000	
	F_S2_	1.782	1.584	0.631	24.870	1.271	0.802	1.650	1.109	1.109	
	F_S3_	1.579	1.470	0.680	24.480	1.295	0.881	2.531	0.203	1.312	
	F_S4_	2.131	1.771	0.565	24.640	1.271	0.718	3.248	0.552	1.864	
	F_S5_	1.465	2.028	0.493	–	–	–	–	0.666	2.530	0.779
Hucnucma	F_S0_	0.343	1.169	0.856	–	–	–	–	–	–	
	F_S1_	0.253	1.376	0.727	24.730	1.271	0.924	0.924		0.000	
	F_S2_	0.334	0.964	1.037	24.670	1.271	1.318	2.242	0.081	0.081	
	F_S3_	1.145	1.005	0.995	24.400	1.295	1.289	3.531	0.811	0.892	
	F_S4_	1.160	4.308	0.232	24.480	1.295	0.301	3.831	0.015	0.907	
	F_S5_	0.751	1.987	0.503	–	–	–	–	0.409	1.316	0.343
Hoctun	F_S0_	0.484	1.633	0.612	–	–	–	–	–	–	
	F_S1_	0.231	1.358	0.736	24.780	1.271	0.936	0.936		0.000	
	F_S2_	0.484	0.889	1.125	24.620	1.271	1.430	2.366	0.253	0.253	
	F_S3_	0.573	1.348	0.742	24.460	1.295	0.961	3.326	0.089	0.342	
	F_S4_	0.754	1.764	0.567	24.860	1.271	0.721	4.047	0.181	0.523	
	F_S5_	0.949	1.479	0.676	–	–	–	–	0.195	0.718	0.177
Agua Dulce	F_S0_	0.802	3.734	0.268	–	–	–	–	–	–	
	F_S1_	1.066	4.157	0.241	24.730	1.271	0.306	0.306		0.000	
	F_S2_	1.081	3.589	0.279	24.850	1.271	0.354	0.660	0.015	0.015	
	F_S3_	1.064	3.321	0.301	24.290	1.295	0.390	1.050	0.017	0.032	
	F_S4_	1.146	2.929	0.341	24.540	1.271	0.434	1.484	0.082	0.114	
	F_S5_	1.098	3.530	0.283	–	–	–	–	0.048	0.162	0.109
Jose Cardel	F_S0_	1.267	4.018	0.249	–	–	–	–	–	–	
	F_S1_	1.478	2.152	0.465	24.210	1.295	0.602	0.602		0.000	
	F_S2_	1.631	2.655	0.377	24.370	1.295	0.488	1.090	0.153	0.153	
	F_S3_	1.686	2.486	0.402	22.480	1.346	0.541	1.631	0.055	0.208	
	F_S4_	1.981	1.808	0.553	24.130	1.295	0.716	2.347	0.295	0.503	
	F_S5_	2.017	2.120	0.472	–	–	–	–	0.036	0.539	0.230

**Table 5:** Estimation of heritability (*h*^2^) of resistance in *Aedes aegypti* after deltamethrin selection for lethal concentration (LC_50_)

Population	Generation	LC_50_	b	δt	Pt	it	S	ΣS	R	ΣR	*h*^2^
Merida	F_S0_	5.350	1.066	0.938	–	–	–	–	–	–	
	F_S1_	5.600	1.827	0.547	22.130	1.346	0.737	0.737		0.000	
	F_S2_	5.800	1.824	0.548	23.990	1.295	0.710	1.447	0.200	0.200	
	F_S3_	7.870	1.930	0.518	20.710	1.400	0.725	2.172	2.070	2.270	
	F_S4_	7.730	1.926	0.519	24.120	1.295	0.672	2.844	0.140	2.410	
	F_S5_	7.880	1.824	0.548	–	–	–	–	0.150	2.560	0.900
Progreso	F_S0_	0.749	0.979	1.021	–	–	–	–	–	–	
	F_S1_	1.060	1.107	0.904	24.79	1.271	1.149	1.149		0.000	
	F_S2_	1.715	1.532	0.653	24.860	1.271	0.830	1.978	0.655	0.655	
	F_S3_	2.378	1.654	0.605	24.460	1.295	0.783	2.761	0.663	1.318	
	F_S4_	2.063	2.186	0.457	24.580	1.271	0.581	3.343	0.315	1.633	
	F_S5_	1.663	2.008	0.498	–	–	–	–	0.400	2.033	0.608
Hucnucma	F_S0_	0.245	1.244	0.804	–	–	–	–	–	–	
	F_S1_	0.694	1.393	0.718	24.680	1.271	0.912	0.912		0.000	
	F_S2_	0.805	1.458	0.686	24.670	1.271	0.872	1.784	0.111	0.111	
	F_S3_	1.164	1.940	0.516	24.240	1.295	0.668	2.452	0.359	0.470	
	F_S4_	1.331	2.453	0.408	24.480	1.295	0.528	2.980	0.167	0.637	
	F_S5_	1.370	1.922	0.520	–	–	–	–	0.039	0.676	0.227
Hoctun	F_S0_	1.302	1.928	0.519	–	–	–	–	–	–	
	F_S1_	2.501	2.239	0.447	24.79	1.271	0.568	0.568		0.000	
	F_S2_	3.270	2.676	0.374	23.830	1.271	0.475	1.042	0.769	0.769	
	F_S3_	2.764	2.521	0.397	24.410	1.295	0.514	1.556	0.506	1.275	
	F_S4_	2.677	2.472	0.405	24.890	1.271	0.514	2.070	0.087	1.362	
	F_S5_	2.076	2.950	0.339	–	–	–	–	0.601	1.963	0.948
Agua Dulce	F_S0_	1.000	2.278	0.439	–	–	–	–	–	–	
	F_S1_	1.410	2.741	0.365	24.77	1.271	0.464	0.464		0.000	
	F_S2_	2.023	2.190	0.457	24.820	1.271	0.580	1.044	0.613	0.613	
	F_S3_	2.120	2.058	0.486	24.280	1.295	0.629	1.674	0.097	0.710	
	F_S4_	2.035	2.285	0.438	24.520	1.271	0.556	2.230	0.085	0.795	
	F_S5_	1.957	1.855	0.539	–	–	–	–	0.078	0.873	0.392
José Cardel	F_S0_	1.638	1.895	0.528	–	–	–	–	–	–	
	F_S1_	2.305	2.617	0.382	24.210	1.295	0.495	0.495		0.000	
	F_S2_	2.436	2.429	0.412	24.370	1.295	0.533	1.028	0.131	0.131	
	F_S3_	2.356	2.827	0.354	22.640	1.320	0.467	1.495	0.080	0.211	
	F_S4_	2.260	2.814	0.355	24.130	1.295	0.460	1.955	0.096	0.307	
	F_S5_	2.849	3.346	0.299	–	–	–	–	0.589	0.896	0.458

### Molecular assays

Tables 6, 7, 8 summarize the frequency of L410, I1016 and C1534 alleles across all populations and generations (F_S0_–F_S5_). The three *kdr* mutations were present in all the parental populations, with frequencies ranging between 0.36–078 for the L410 allele and 0.34–0.77 for I1016. The highest frequencies were for C1534, ranging from 0.59–1. All populations were in Hardy-Weinberg equilibrium at the parental generation (F_S0_), with the exception of Jose Cardel for the mutation F1534C.

**Table 6:** V410L genotypes and allele frequencies in *Aedes aegypti* females in the parental and all selected generations. *χ*^2^ Hardy-Weinberg, inbreeding coefficients (F_IS_) and significance testing was calculated for the parental generation

Population	Generation	*n*	VV	VL	LL	Freq. (95% CI)	F_IS_	*χ*^2^ Hardy- Weinberg	*P-*value
Merida	F_S0_	32	3	8	21	0.78 (0.60–0.89)	0.26	2.3	0.128
	F_S1_	29	5	14	10	0.59 (0.40–0.74)	–	–	
	F_S2_	32	4	11	17	0.70 (0.52–0.83)	–	–	
	F_S3_	29	0	5	24	0.91 (0.74–0.98)	–	–	
	F_S4_	32	1	8	23	0.84 (0.67–0.93)	–	–	
	F_S5_	32	0	5	27	0.92 (0.76–0.98)	–	–	
Progreso	F_S0_	30	4	13	13	0.65 (0.47–0.79)	0.04	0.06	0.794
	F_S1_	29	0	5	24	0.91 (0.75–0.98)	–	–	
	F_S2_	30	1	5	24	0.88 (0.71–0.96)	–	–	
	F_S3_	32	1	2	29	0.94 (0.78–0.99)	–	–	
	F_S4_	30	1	12	17	0.77 (0.58–0.88)	–	–	
	F_S5_	32	0	5	27	0.92 (0.76–0.98)	–	–	
Hunucma	F_S0_	32	14	13	5	0.36 (0.21–0.53)	0.11	0.44	0.505
	F_S1_	32	7	13	12	0.58 (0.40–0.73)	–	–	
	F_S2_	21	6	9	6	0.50 (0.30–0.69)	–	–	
	F_S3_	30	2	14	14	0.70 (0.51–0.83)	–	–	
	F_S4_	32	1	3	28	0.92 (0.76–0.98)	–	–	
	F_S5_	30	0	3	27	0.95 (0.79–0.99)	–	–	
Hoctun	F_S0_	32	6	21	5	0.48 (0.32–0.64)	-0.31	3.15	0.075
	F_S1_	31	15	11	5	0.34 (0.19–0.51)	–	–	
	F_S2_	28	4	13	11	0.63 (0.44–0.77)	–	–	
	F_S3_	31	0	4	27	0.94 (0.78–0.99)	–	–	
	F_S4_	32	0	9	23	0.86 (0.69–0.94)	–	–	
	F_S5_	30	0	8	22	0.87 (0.69–0.95)	–	–	
Agua Dulce	F_S0_	30	6	15	9	0.55 (0.37–0.71)	-0.01	0.003	0.956
	F_S1_	30	3	13	14	0.68 (0.50–0.82)	–	–	
	F_S2_	27	8	14	5	0.44 (0.27–0.62)	–	–	
	F_S3_	34	6	22	6	0.50 (0.34–0.65)	–	–	
	F_S4_	32	0	5	27	0.92 (0.76–0.98)	–	–	
	F_S5_	32	0	1	31	0.98 (0.84–1.00)	–	–	
Jose Cardel	F_S0_	32	4	14	14	0.66 (0.48–0.79)	0.03	0.02	0.863
	F_S1_	32	3	11	18	0.73 (0.55–0.85)	–	–	
	F_S2_	32	0	7	25	0.89 (0.72–0.96)	–	–	
	F_S3_	30	0	7	23	0.88 (0.71–0.96)	–	–	
	F_S4_	32	0	4	28	0.94 (0.78–0.99)	–	–	
	F_S5_	30	1	10	19	0.80 (0.62–0.90)	–	–	

*Abbreviations*: n, sample size; V410/V410, wild type; V410/L410, heterozygotes; L410/L410, homozygotes resistant

**Table 7:** V1016I genotypes and allele frequencies in *Aedes aegypti* females in the parental and all selected generations. *χ*^2^ Hardy-Weinberg, inbreeding coefficients (F_IS_) and significance testing was calculated for the parental generation

Population	Generation	*n*	VV	VI	II	Freq. (95% CI)	F_IS_	*χ*^2^ Hardy- Weinberg	*P-*value
Merida	F_S0_	32	3	9	20	0.77 (0.59–0.88)	0.21	1.49	0.221
	F_S1_	30	5	15	10	0.58 (0.40–0.73)	–	–	
	F_S2_	32	4	10	18	0.72 (0.54–0.84)	–	–	
	F_S3_	31	0	7	24	0.89 (0.72–0.96)	–	–	
	F_S4_	32	0	9	23	0.86 (0.69–0.94)	–	–	
	F_S5_	32	0	5	27	0.92 (0.76–0.98)	–	–	
Progreso	F_S0_	32	4	12	16	0.69 (0.51–0.82)	0.12	0.51	0.472
	F_S1_	32	0	22	10	0.66 (0.48–0.79)	–	–	
	F_S2_	30	1	5	24	0.88 (0.71–0.96)	–	–	
	F_S3_	32	1	3	28	0.92 (0.76–0.98)	–	–	
	F_S4_	32	1	12	19	0.78 (0.60–0.89)	–	–	
	F_S5_	31	0	5	26	0.92 (0.76–0.98)	–	–	
Hunucma	F_S0_	32	14	14	4	0.34 (0.29–0.51)	0.03	0.02	0.863
	F_S1_	32	7	14	11	0.56 (0.39–0.71)	–	–	
	F_S2_	32	7	19	6	0.48 (0.32–0.64)	–	–	
	F_S3_	29	2	15	12	0.67 (0.48–0.81)	–	–	
	F_S4_	32	1	2	29	0.94 (0.78–0.99)	–	–	
	F_S5_	31	0	3	28	0.95 (0.80–0.99)	–	–	
Hoctun	F_S0_	32	7	21	4	0.45 (0.29–0.62)	−0.32	3.36	0.066
	F_S1_	32	16	11	5	0.33 (0.19–0.50)	–	–	
	F_S2_	27	4	14	9	0.59 (0.40–0.75)	–	–	
	F_S3_	30	0	5	25	0.92 (0.75–0.98)	–	–	
	F_S4_	32	0	10	22	0.84 (0.67–0.93)	–	–	
	F_S5_	32	0	8	24	0.88 (0.71–0.95)	–	–	
Agua Dulce	F_S0_	32	6	16	10	0.56 (0.39–0.71)	-0.01	0.01	0.928
	F_S1_	32	4	14	14	0.66 (0.48–0.79)	–	–	
	F_S2_	31	8	18	5	0.45 (0.29–0.62)	–	–	
	F_S3_	34	2	21	11	0.63 (0.46–0.77)	–	–	
	F_S4_	32	0	4	28	0.94 (0.78–0.99)	–	–	
	F_S5_	32	0	2	30	0.96 (0.82–1.00)	–	–	
Jose Cardel	F_S0_	31	5	13	13	0.63 (0.45–0.77)	0.1	0.31	0.572
	F_S1_	30	7	8	15	0.63 (0.45–0.78)	–	–	
	F_S2_	32	0	8	24	0.88 (0.71–0.95)	–	–	
	F_S3_	30	0	17	13	0.72 (0.53–0.84)	–	–	
	F_S4_	32	0	3	29	0.95 (0.80–0.99)	–	–	
	F_S5_	31	0	5	26	0.92 (0.76–0.98)	–	–	

*Abbreviations*: n, sample size; V1016/V1016, wild type; V1016/I1016, heterozygotes; I1016/I1016, homozygotes resistant

**Table 8:** F1534C genotypes and allele frequencies in *Aedes aegypti* females in the parental and all selected generations. *χ*^2^ Hardy-Weinberg, inbreeding coefficients (F_IS_) and significance testing was calculated for the parental generation

Population	Generation	n	FF	FC	CC	Freq. (95% CI)	F_IS_	*χ*^2^ Hardy- Weinberg	*P-*value
Merida	F_S0_	32	0	0	32	1.00 (0.86–1.00)	–	–	–
	F_S1_	32	0	0	32	1.00 (0.86–1.00)	–	–	
	F_S2_	32	0	0	32	1.00 (0.86–1.00)	–	–	
	F_S3_	31	0	0	31	1.00 (0.86–1.00)	–	–	
	F_S4_	32	0	0	32	1.00 (0.86–1.00)	–	–	
	F_S5_	32	0	0	32	1.00 (0.86–1.00)	–	–	
Progreso	F_S0_	32	0	2	30	0.97 (0.82–1.00)	−0.03	0.03	0.855
	F_S1_	32	0	0	32	1.00 (0.86–1.00)	–	–	
	F_S2_	30	0	0	30	1.00 (0.86–1.00)	–	–	
	F_S3_	32	0	0	32	1.00 (0.86–1.00)	–	–	
	F_S4_	32	0	0	32	1.00 (0.86–1.00)	–	–	
	F_S5_	32	0	0	32	1.00 (0.86–1.00)	–	–	
Hunucma	F_S0_	32	5	13	14	0.64 (0.46–0.78)	0.11	0.44	0.505
	F_S1_	32	2	6	24	0.84 (0.67–0.93)	–	–	
	F_S2_	32	5	4	23	0.78 (0.60–0.89)	–	–	
	F_S3_	29	2	7	20	0.81 (0.62–0.91)	–	–	
	F_S4_	32	0	0	32	1.00 (0.86–1.00)	–	–	
	F_S5_	32	0	0	32	1.00 (0.86–1.00)	–	–	
Hoctun	F_S0_	32	4	18	10	0.59 (0.42–0.74)	0.16	0.88	0.347
	F_S1_	32	7	11	14	0.61 (0.43–0.75)	–	–	
	F_S2_	29	3	5	21	0.81 (0.62–0.91)	–	–	
	F_S3_	30	0	0	30	1.00 (0.86–1.00)	–	–	
	F_S4_	32	0	0	32	1.00 (0.86–1.00)	–	–	
	F_S5_	32	0	0	32	1.00 (0.86–1.00)	–	–	
Agua Dulce	F_S0_	32	0	0	32	1.00 (0.86–1.00)	–	–	–
	F_S1_	32	0	0	32	1.00 (0.86–1.00)	–	–	
	F_S2_	31	0	0	31	1.00 (0.86–1.00)	–	–	
	F_S3_	34	0	0	34	1.00 (0.86–1.00)	–	–	
	F_S4_	32	0	0	32	1.00 (0.86–1.00)	–	–	
	F_S5_	32	0	0	32	1.00 (0.86–1.00)	–	–	
Jose Cardel	F_S0_	31	2	3	26	0.89 (0.72–0.96)	0.51	8.28	0.004
	F_S1_	32	0	0	32	1.00 (0.86–1.00)	–	–	
	F_S2_	32	0	0	32	1.00 (0.86–1.00)	–	–	
	F_S3_	30	0	0	30	1.00 (0.86–1.00)	–	–	
	F_S4_	32	0	0	32	1.00 (0.86–1.00)	–	–	
	F_S5_	32	0	0	32	1.00 (0.86–1.00)	–	–	

*Abbreviations*: n, sample size; F1534/F1534, wild type; F1534/C1534, heterozygotes; C1534/C1534, homozygotes resistant

**Fig. 3:**
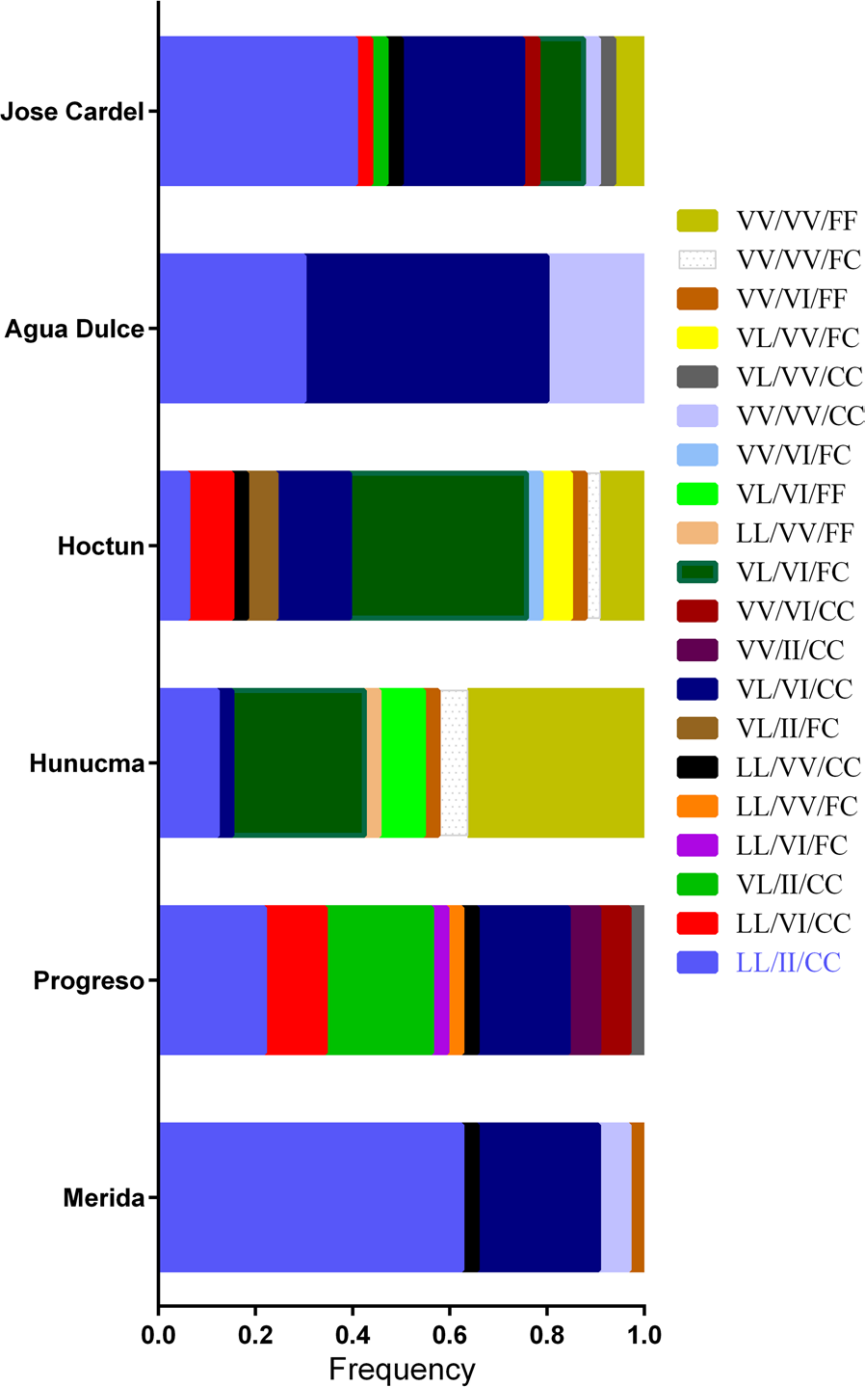
Frequencies of the tri-locus genotypes in the parental generation (F_S0_) of each population of *Aedes aegypti.* Genotypes order: 410/1016/1534

**Fig. 4:**
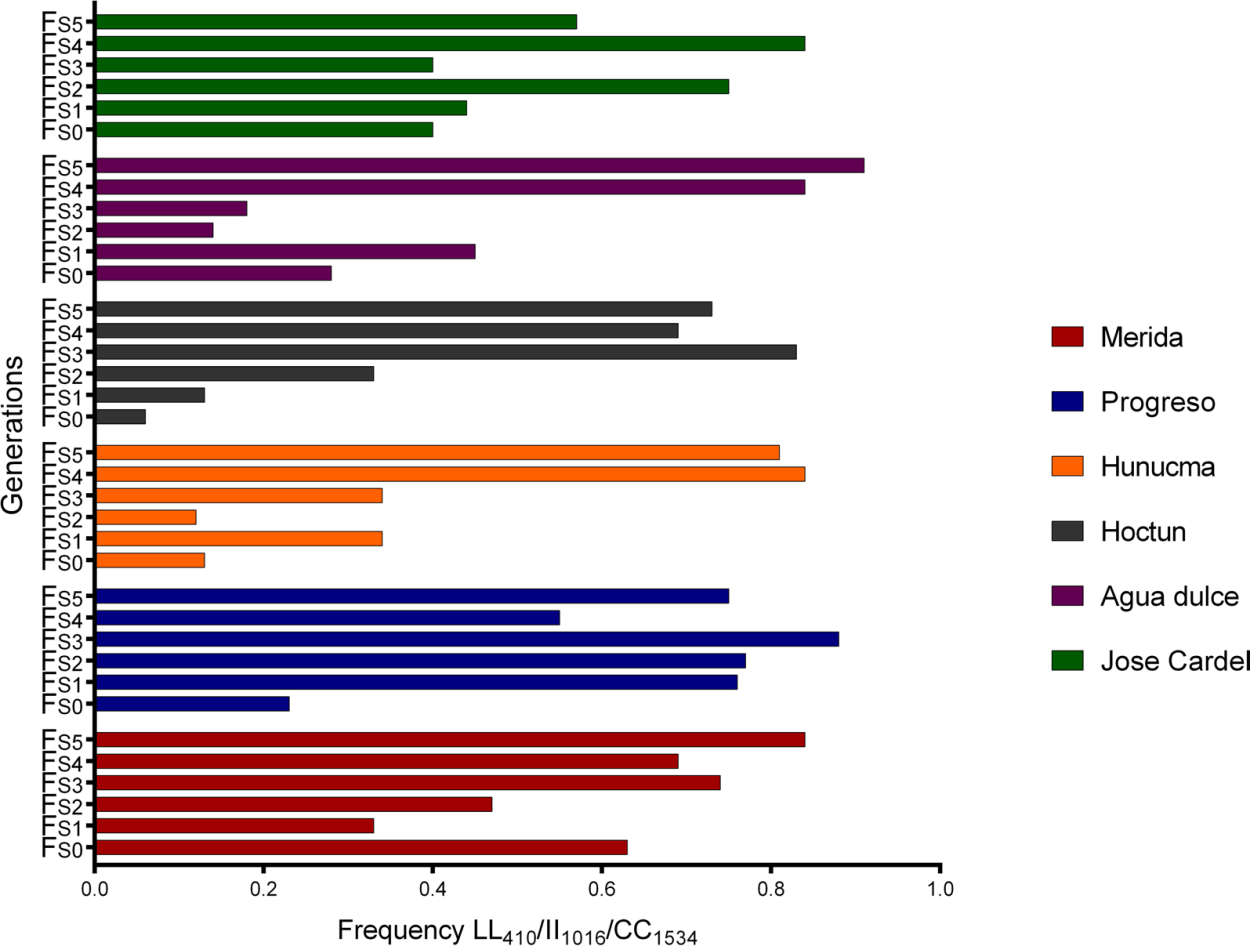
Frequencies of the resistant trilocus genotype LL_410_/II_1,016_/CC_1,534_ across selection with deltamethrin (F_S0_-F_S1_) from six populations of *Aedes aegypti* from Mexico

A total of 20 combinations of tri-locus genotypes were detected among the populations in the parental generation (F_S0_). Figure 3 shows the frequency of each of the 20 tri-locus genotype combinations. Triple homozygote resistant genotype (LL_410_/II_1016_/CC_1534_) occurred in all parental (F_S0_) populations of *Ae. aegypti* with the highest frequency for the Merida population (0.63) and the lowest for Hoctun (0.06), and their frequency increased as generations were subsequently selected (Fig. 4).

Interestingly, the triple heterozygous genotype (VL_410_/VI_1016_/FC_1534_) was only found in the populations of Hunucma, Hoctu and Jose Cardel, in which the triple wild-type genotype (VV_410_/VI_1016_/FC_1534_) was also found.

In general, the frequencies of the three *kdr* alleles increased over the selected generations. The frequencies of L410 from the parental generation to the last selected generation increased from 0.78 to 0.92 in the Merida population, 0.65 to 0.92 in Progreso, 0.36 to 0.95 in Hunucma, 0.48 to 0.87 in Hoctun, 0.55 to 0.98 in Agua Dulce and 0.66 to 0.80 in Jose Cardel.

For the I1016 allele, increases of 0.77–0.92 were observed in the Merida population, 0.69–0.92 in Progreso, 0.45–0.95 in Hunucma, 0.34–0.88 in Hoctun, 0.56–0.96 in Agua Dulce and 0.63–0.92 in Jose Cardel.

The frequencies of the C1534 allele increased from 0.59 to 1 in the Hoctun population, 0.64 to 1 in Hunucma, 0.89 to 1 in Jose Cardel and 0.97 to 1 in Progreso; the C1534 allele was fixed at 1.0 in the Merida and Agua Dulce populations in all generations. This allele reached fixation after the first selection with deltamethrin (F_S1_) in the population from Progreso and Jose Cardel, in the third selected generation for Hoctun and in the fourth selected generation for Hunucma.

**Fig. 5:**
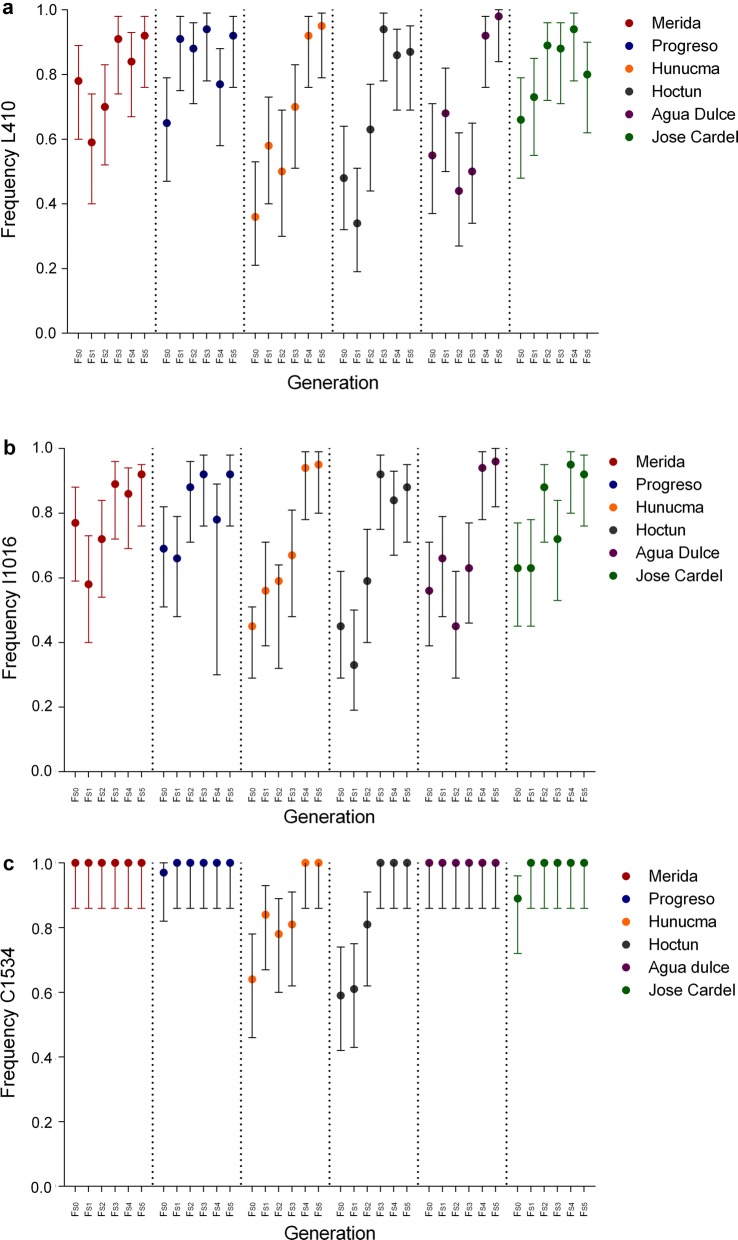
Allele frequencies of L410 (**a**), I1016 (**b**) and C1534 (**c**) and their maximum and minimum frequencies in six *Aedes aegypti* populations across selection with deltamethrin (F_S0_-F_S5_)

**Fig. 6:**
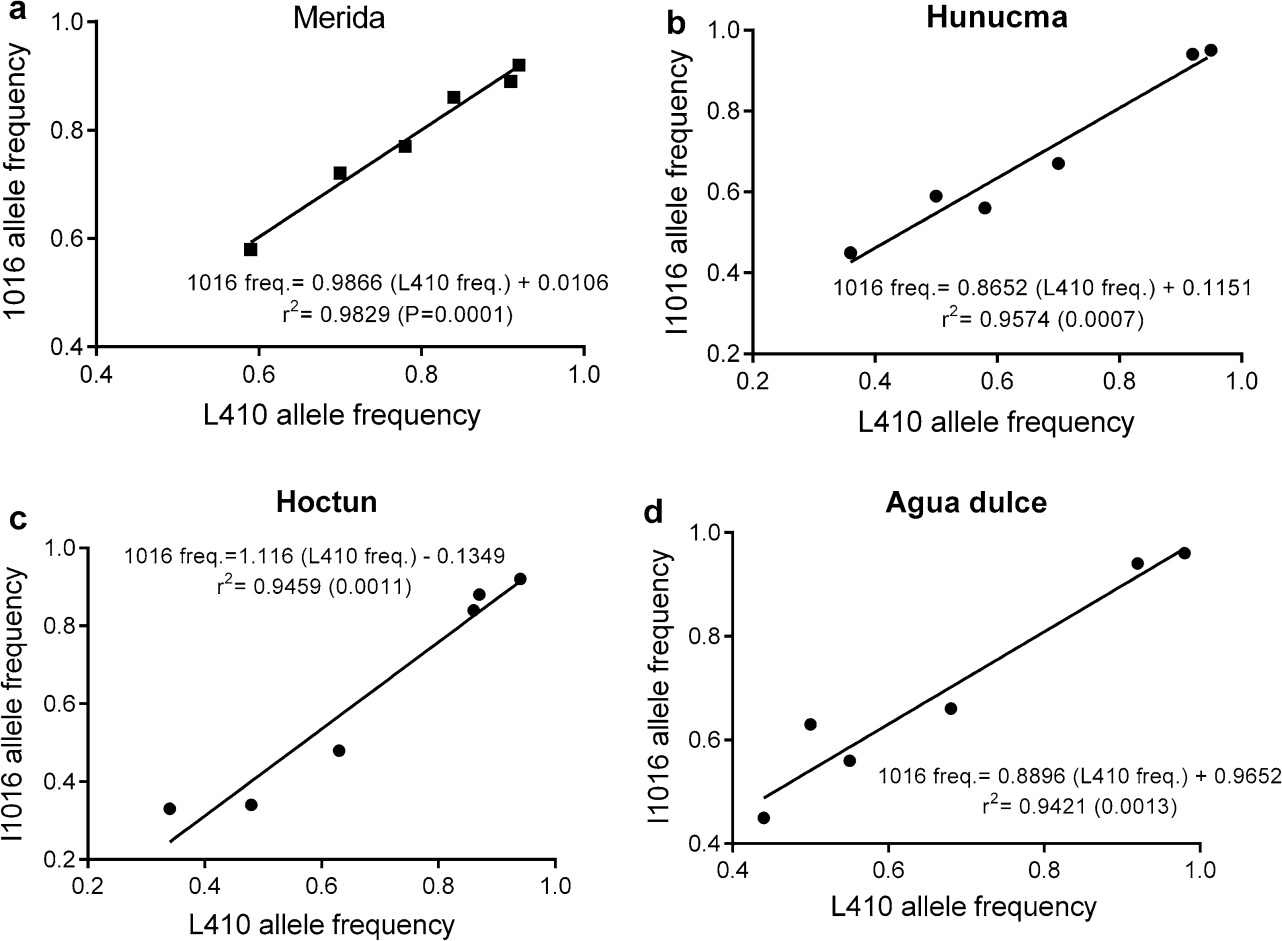
Regression analysis of allele frequencies of two *para* mutations: I1016 *vs* L410 across the selection with deltamethrin (F_S0_-F_S5_) in *Aedes aegypti* from Merida (**a**), Hunucma (**b**), Hoctun (**c**), Agua dulce (**d**)

**Fig. 7:**
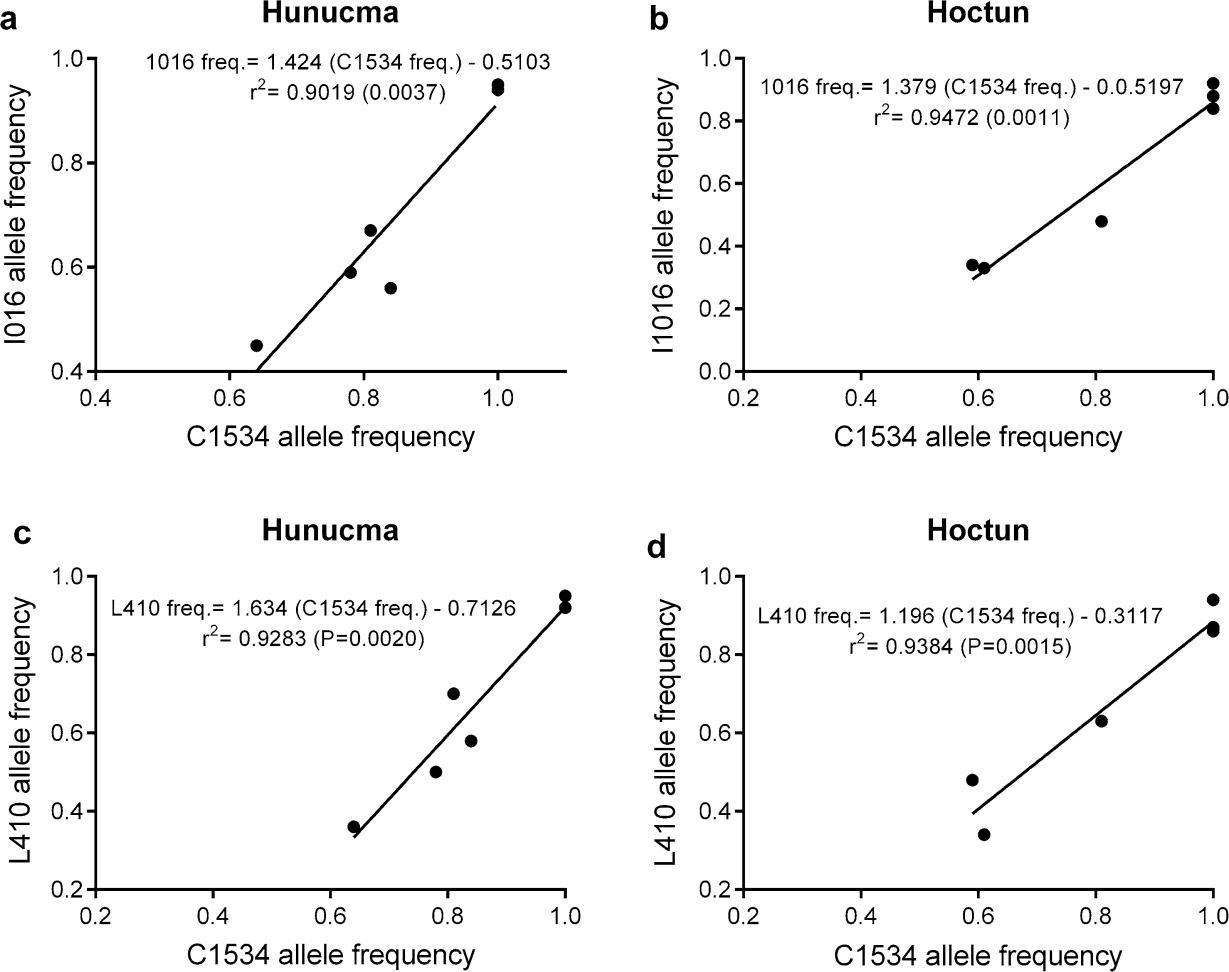
Regression analysis of allele frequencies of C1534 *vs* I016 (**a**, **b**) and C1534 *vs* L410 (**b**, **c**) across the selection with deltamethrin (F_S0_-F_S5_) in *Aedes aegypti* from Hunucma and Hoctun

The L410 and I1016 alleles were selected in almost the same proportion from F_S0_ to the F_S5_ in all populations analyzed (Tables 6, 7, Fig. 5). Figure 6 shows that the increase in the frequencies of the L410 alleles in response to the selection with deltamethrin correlates significantly (*P *< 0.05) with the increase in the frequencies of the I106 allele by 98% in the population of Merida, 96% in Hunucma, 95% in Hoctun and 95% in Agua Dulce. Additionally, in the populations of Hoctun and Hunucma we found that the allelic frequency of C1534 increased significantly (*P* < 0.05) with the increase in allele frequency of I1016 and L410 throughout the selection in the populations of Hunucma and Hoctun (Fig. 7).

The frequency of the mutant alleles for each mutation was correlated with the KC_50_ and LC_50_ values across the generations of selection (Table 9). KC_50_ values were significantly correlated with L410 frequencies in the Merida and Hoctun populations (*P *< 0.05); and with the frequencies of I1016 in the Merida, Hoctun and Jose Cardel (*P *< 0.05). Meanwhile, the frequencies of L410 and I1016 were significantly correlated with LC_50_ values in the Merida and Hunucma populations as well as C1534 correlated with LC_50_ values in Hunucma and Jose Cardel (*P *< 0.05). The presence and frequency of triple homozygous mutant individuals showed a significant correlation with KC_50_ only in the Merida population and LC_50_ in the Hunucma population (*P *< 0.05).

**Table 9:** Pearsonʼs correlation coefficients (*r*) (*P-*values in parenthesis) between the resistant allelic frequencies for L410, I1016, C1534 and with the frequencies of tri-locus genotypes *vs* KC_50_ and LC_50_ across the generations of selection with deltamethrin in five populations of *Ae. aegypti* from Mexico

KC_50_/LC_50_	Populations					
	Merida	Progreso	Hunucma	Hoctun	Agua dulce	Jose Cardel
L410						
KC_50_	0.8303 (*P *= 0.040)	ns	ns	0.8090 (*P *= 0.050)	ns	ns
LC_50_	0.8323 (*P *= 0.039)	ns	0.9506 (*P *= 0.004)	ns	ns	ns
I1016						
KC_50_	0.8459 (*P *= 0.033)	ns	ns	0.8305 (*P *= 0.040)	ns	0.8824 (*P *= 0.020)
LC_50_	0.8410 (*P *= 0.036)	ns	0.9249 (*P *= 0.008)	ns	ns	ns
C1534						
KC_50_	–	ns	ns	ns	–	ns
LC_50_	–	ns	0.9023 (*P *= 0.014)	ns	–	0.8401 (*P *= 0.036)
LL+II+CC						
KC_50_	0.8678 (*P *= 0.0251)	ns	ns	ns	ns	ns
LC_50_	–	ns	0.8255(*P *= 0.043)	ns	ns	ns

*Abbreviation*: ns, not significant

### Biochemical assays

Table 10 shows the mean enzyme activity levels detected in the biochemical assays in the parental generation and all deltamethrin-selected generations, where at least 30 individuals per population were analyzed. When comparing values from the parental generations with the 99th percentile of the New Orleans reference strain, five of the six populations analyzed (Merida, Progreso, Hunucma, Jose Cardel and Agua Dulce) showed altered enzyme activity for α-esterases (˃ 50% of the individuals exceeded the threshold of resistance), and Hoctun showed incipient altered enzyme activity (39% of the individuals exceeded the threshold). Merida, Agua Dulce and Jose Cardel showed altered enzyme activity for β-esterases (˃ 50%), and Progreso, Hunucma and Hoctun showed incipient enzyme alteration with percentages ranging between 16–43%. Altered activity of MFOs was detected in Progreso, Hoctun, Agua Dulce and José Cardel in 69–95% of the specimens analyzed. Only the Agua Dulce population showed altered enzyme activity for GSTs (71%) in the parental generation.

**Table 10:** Quantification of enzymatic activity in *Aedes aegypti* populations in the parental generation and deltamethrin-selected generations with respect to the New Orleans reference strain

Population/generation		α-esterases			β-esterases		MFO		GST	
		(nmol/mg ptn/min)			(nmol/mg ptn/min)		(µg Cyt/mg de ptn)		(nmol/mg ptn/min)	
		*n*	Mean ± SD	%>p99^c^	Mean ± SD	%>p99	Mean ± SD	%>p99	Mean ± SD	%>p99
	New Orleans^a^	30	3.53 ± 0.50	4.77^b^	8.56 ± 0.97	10.9^b^	72.59 ± 9.63	94.31^b^	0.05 ± 0.01	0.09^b^
F_0_	Merida	30	7.18 ± 1.21	**99**	13. 11 ± 2.26	**79**	87.30 ± 16.44	31	0.03 ± 0.01	0
	Progreso	30	5.31 ± 0.64	**82**	10.00 ± 1.03	16	113.2 ± 22.45	**69**	0.08 ± 0.03	48
	Hunucma	30	6. 23 ± 0.73	**99**	10.95 ± 0.80	43	85.08 ± 12.84	23	0.05 ± 0.01	1
	Hoctún	30	4.80 ± 0.69	39	10.76 ± 1.10	40	106.20 ± 11.04	**81**	0.07 ± 0.01	8
	Agua dulce	30	6.58 ± 0.85	**100**	11.11 ± 0.86	**54**	132.10 ± 30.02	**97**	0.11 ± 0.03	**71**
	Cardel	30	7.77 ± 0.88	**100**	12.75 ± 1.56	**95**	128.10 ± 16.51	**95**	0.06 ± 0.01	3
F_1_	Merida	30	5.73 ± 0.69	**88**	10.33 ± 0.78	24	94.27 ± 10.83	50	0.47 ± 0.12	0
	Progreso	30	7.86 ± 0.64	**100**	9.81 ± 1.12	11	98.54 ± 13.80	**56**	0.63 ± 0.27	18
	Hunucma	30	7.80 ± 0.84	**100**	10.02 ± 1.28	18	112.57 ± 26.94	**70**	0.89 ± 0.28	**68**
	Hoctún	30	4.04 ± 0.84	11	9.43 ± 1.71	15	89.01 ± 18.30	38	0.34 ± 0.37	1
	Agua dulce	30	6.69 ± 0.78	**100**	11. 35 ± 1.69	**57**	107.46 ± 13.62	**87**	0.11 ± 0.64	6
	Cardel	30	3.80 ± 0.88	10	8.11 ±1.07	0	96.22 ± 15.30	50	0.87 ± 0.30	**50**
F_2_	Merida	30	6.75 ± 0.60	**99**	11.23 ± 1.26	**74**	91.57 ± 11.82	33	0.70 ± 0.15	25
	Progreso	30	6.52 ± 0.87	**99**	12.44 ± 1.30	**89**	87.45 ± 14.30	32	0.54 ± 0.18	3
	Hunucma	30	6.03 ± 0.82	**96**	10.51 ± 0.66	**79**	82.38 ± 7.83	7	0.37 ± 0.11	0
	Hoctún	30	6.27 ± 0.47	**99**	10.84 ± 0.98	44	86.52 ± 12.45	31	0.87 ± 0.27	**58**
	Agua dulce	30	6.69 ± 0.79	**99**	9.45 ± 1.16	5	81.67 ± 11.91	6	0.73 ± 0.29	31
	Cardel	30	6.60 ± 0.65	**99**	12.87 ± 1.15	**94**	95.78 ± 14.69	39	0.32 ± 0.11	0
F_3_	Merida	30	4.43 ± 5.22	**64**	9.93 ± 1.16	20	79.41 ± 12.43	10	0.56 ± 0.21	17
	Progreso	30	6.97 ± 0.45	**99**	11.70 ± 0.93	**81**	84.93 ± 7.03	9	1.13 ± 0.26	**90**
	Hunucma	30	8.80 ± 0.56	**99**	13.85 ± 1.05	**99**	76.67 ± 7.06	0	0.54 ± 0.12	3
	Hoctún	30	6.21 ± 0.39	**99**	10.50 ± 0.69	33	73.27 ± 4.90	0	0.42 ± 0.11	0
	Agua dulce	30	6.51 ± 0.85	**99**	10.89 ± 1.67	44	83.45 ± 10.15	5	0.13 ± 0.03	0
	Cardel	30	7.21 ± 0.60	**99**	11.17 ± 0.90	49	77.59 ± 6.37	0	0.50 ± 0.14	1
F_4_	Merida	30	4.59 ± 0.67	29	10.19 ± 1.78	20	87.68 ± 8.14	22	0.90 ± 0.21	**75**
	Progreso	30	3.20 ± 0.38	0	6.72 ± 0.60	0	95.73 ± 6.40	**57**	0.61 ± 0.52	34
	Hunucma	30	5.57 ± 0.69	**90**	8.51 ± 0.88	0	84.16 ± 10.74	19	0.64 ± 0.25	22
	Hoctún	30	5.34 ± 0.61	**82**	10.17 ± 0.90	25	82.48 ± 8.22	19	0.49 ± 0.16	1
	Agua dulce	30	5.55 ± 0.57	**94**	9.52 ± 0.86	2	75.84 ± 8.56	0	0.50 ± 0.18	3
	Cardel	30	9.45 ± 0.83	**99**	14.67 ± 1.32	**97**	70.20 ± 9.79	0	0.65 ± 0.23	10
F_5_	Merida	30	4.29 ± 0.55	14	7.53 ± 0.97	0	61.85 ± 8.80	0	0.59 ± 0.18	17
	Progreso	30	5.84 ± 0.68	**92**	12.22 ± 1.17	**91**	72.17 ± 7.59	0	0.78 ± 0.30	47
	Hunucma	30	7.05 ± 0.55	**99**	10.76 ± 0.58	34	70.72 ± 7.03	0	0.45 ± 0.11	0
	Hoctún	30	3.68 ± 0.50	1	8.19 ± 1.19	0	71.93 ± 6.28	0	0.86 ± 0.20	**54**
	Agua dulce	30	6.69 ± 0.81	**99**	11.28 ± 1.40	**55**	67.35 ± 6.25	0	1.03 ± 0.35	**80**
	Cardel	30	4.78 ± 0.81	49	10.27 ± 1.22	29	77.07 ± 6.16	0	0.49 ± 0.17	2

*Abbreviations*: n, sample size; SD, standard deviation

^a^ New Orleans: susceptible reference strain

^b^ p99, 99th percentile for reference strains

^c^%>p99 percentage of individuals above 99th percentile of reference strain, values classified as altered (> 50%) are in bold

The results show variations in enzyme activity across populations over the generations post-selection. However, when comparing F_S5_*vs* F_S0_, α-esterases remained altered in the Progreso, Hunucma and Agua Dulce; β-esterases in Progreso and Agua Dulce, and an increase in the mean activity levels of GSTs was observed only in the Hoctun and Agua Dulce populations (*P *< 0.0001) (Table 10). GSTs were the only enzyme family for which a significant correlation was detected between level of activity and LC_50_ values, but only in the F_S4_ generation (*r *= 0.83, *P* < 0.05).

## Discussion

Target-site insensitivity and increased metabolic activity are key mechanisms of pyrethroid resistance in mosquito populations worldwide. The control of adult mosquitoes in Mexico has been carried out mainly with pyrethroids since 1999, and deltamethrin has been in use since the beginning of 2000 [9]. In the present study, five of the populations analyzed were found to be highly resistant to the pyrethroid deltamethrin in the parental generation according to the LC_50_ (RRLC_50_ > 10-fold), and only one population showed moderate resistance (RRCL_50_ 5–10-fold). Likewise, high RRKC_50_ values were detected in two populations, three showed moderate resistance, and one showed low resistance. These results suggest that deltamethrin resistance is well-established in these populations.

In response to further deltamethrin selection, both LC_50_ and KC_50_ increased over the generations, with the concentrations capable of knocking down or killing 50% of the population at least doubling by F_S5_ compared to F_S0_. The highest heritability values (h^2^ ≥ 0.90) for LC_50_ were detected in the populations from Merida and Hoctun, followed by the Progreso population (0.61), suggesting high values of additive genetic variance. The highest value of h^2^ for KC_50_ was detected in the Progreso population (h^2^ > 0.70), followed by the Merida population (0.52). A high h^2^ value predicts a rapid response to selection, while a low value suggests a slow response to artificial selection [46].

The frequencies of the L410, I1016 and C1534 alleles increased with deltamethrin selection in all populations. When analyzing the frequencies in the last selected generation among the different populations, the frequencies of the L410 allele ranged between 0.80–0.98; this allele is able to significantly decrease the sensitivity of the sodium channel for both permethrin and deltamethrin [27]. The I1016 allele frequencies were 0.88–0.96 in F_S5_ and the C1534 allele reached fixation by F_S5_ in all populations analyzed. These results suggest that deltamethrin can select for C1534 more rapidly than I1016, corroborating the findings of Alvarez et al. [60].

The co-occurrence of I1016 and C1534 and high levels of pyrethroid resistance has been reported in other countries [61–66]. In Mexico, the co-occurrence of these two mutations and pyrethroid resistance has been also documented [34, 50]. Vera-Maloof et al. [34] originally suggested that in order to present resistance to pyrethroids, the sequential evolution of both mutations was necessary. They considered it unlikely that I1016 had evolved independently due to the low fitness exhibited by haplotype I1016/F1534; instead, they hypothesized that C1534 first evolved by conferring a low level of resistance individually and that I1016 arose from haplotype V1016/C1534 and selected quickly due to the high level of resistance conferred by the double mutant haplotype. The sequential selection of F1534C and V1016I was later confirm by Chen et al. [31].

The V410L mutation was reported for the first time in the sodium channels of mosquitoes by Haddi et al. [27]. They demonstrated that this mutation reduced the sensitivity of the *vgsc* gene to both type I (i.e. permethrin) and type II pyrethroids (i.e. deltamethrin). The presence of this mutation has been demonstrated in *Ae. aegypti* from Mexico for more than 16 years and it has been shown that L410 is in greater linkage disequilibrium with I1016 than with C1534. Our results show that L410 and I1016 responded to the selection with deltamethrin in a similar way in populations where C1534 was fixed; or in Hoctun and Hunucma where the increase in C1534 was associated with the increase in L410 and I1016. This is consistent with the proposed sequential model where both V410L and V1016I might have occurred independently on a C1534 haplotype and then became cis to C1534 by recombination. Or alternatively and considering that the F1534C mutation was fixed in practically four of the six basal populations (F_S0_), the three mutations arose independently at very low frequencies, and then by two recombination events, came to occur in a cis arrangement [30].

Increased activity of mixed-function oxidases (MFOs) and esterase activity have been associated with pyrethroid resistance in *Ae. aegypti* [38, 67]. Our results detected incipiently altered levels of MFO activity in four populations. Altered levels of α-esterase activity were detected in five populations, and at least four populations showed altered levels of β-esterase activity. However, there was no association between increased activities of these enzymes and phenotypic resistance, suggesting that these enzymes are not strongly associated with the metabolism of deltamethrin. However, contrary to the findings of Son et al. [68], who reported increased levels of MFOs in response to deltamethrin selection, we found that MFO activity decreased with successive selection. Given that the frequencies of the *kdr* mutations increased significantly with deltamethrin selection, the rapid selection of these mutations could have conditioned the activity of the enzymes in relation to deltamethrin resistance.

One criticism in our methodology is that we compare the enzymatic activity of each generation of deltamethrin in each of the populations with respect to the enzymatic activity of the susceptible New Orleans strain, when the most appropriate comparison should be with respect to the same population, in the same generation, without selection with deltamethrin.

In the metabolic resistance, three families of detoxifying enzymes, oxidases, esterases and glutathione transferases are mainly involved, all associated to confer resistance through overexpression, increased insecticide metabolism or a greater affinity for the chemical [6, 17]. An example of this contribution is reported by Lumjuan et al. [69] who determined an overexpression of GSTe2, GSTe5, GSTe7 and GSTE5-5 of epsilon glutathione transferase in individuals with resistance to DDT and pyrethroids. This fact is directly associated with the reduction in the susceptibility of these individuals to chemical control. Similarly, using quantitative trait loci (QTL) it was shown that temephos resistance is related to a QTL on chromosome two where a carboxylesterase cluster occurs; subsequent studies showed that there is an increase in the expression of these enzymes and therefore a possible intervention as a resistance mechanism [70]. Similar results were reported by Saavedra et al. [71], where the significant participation of an esterase marker discovered by QTL, CCEunk7o, and its relationship to resistance development in conjunction with mutations in the voltage-gated sodium channel was demonstrated.

Alongside gene expression studies, several investigations focus on studying the levels of detoxifying enzymatic activity. Proof of this is the increase in activity suffered by multiple-function oxidases, which includes cytochrome P450, in individuals selected with deltamethrin for 15 generations compared to the unselected susceptible group [60]; however, as for this mechanism to be in itself the one that confers insecticide resistance is questionable since other mechanisms were described for these populations.

To verify that the enzymatic activity has a direct effect on the decrease in susceptibility, synergists are used, chemical compounds that inhibit detoxifying enzymes. By inhibiting the enzymatic activity of multiple function oxidases, esterases and glutathione transferases directly involved as a resistance mechanism, proof of this is reported by Bharati & Saha [72] where an increase in enzymatic activities of multiple-function oxidases (CYP450) and carboxylesterases was identified as a mechanism of resistance, by inhibiting such enzymes, the susceptibility to the pyrethroid deltamethrin and the carbamate propoxur was recovered in populations of *Ae. aegypti* from Bangladesh. A similar pattern was demonstrated in populations of *Ae. aegypti* resistant to DDT and pyrethroids from Selangor, Malaysia, where the inhibition of multiple function oxidases and glutathione-S transferases through an assay with synergists correlated with an increase in the activity of such enzymes as a possible mechanism of resistance [73].

Identifying the direct and exclusive participation of metabolic resistance to insecticides is a complex process due to the simultaneous presence of mechanisms such as target site mutations and elevated levels of detoxifying enzymes. Despite this, the occurrence of *Ae. albopictus* populations free of insecticide-resistant target site mutations has been reported, demonstrating the direct and exclusive participation of cytochrome P450 genes such as CYP6P12 and the overexpression of cuticular genes as the main resistance mechanisms [74].

## Conclusions

The high levels of resistance associated with high frequencies of *kdr* alleles L410, I1016 and C1534 obtained through artificial selection suggest the important role of these mutations in resistance to deltamethrin. The role of metabolic resistance was less clear, as the activity levels of key enzyme groups appeared to increase or decrease without clearly relating to the selection with deltamethrin. These findings highlight the importance of monitoring the susceptibility status of mosquito populations before choosing insecticide products for vector control, especially in areas where resistance may already be present and further selection could quickly result in fixation of *kdr* mutations.

## Data Availability

All data generated or analyzed during this study are included in the published article.

## Abbreviations

*kdr*: knockdown resistance
KC_50_: 50% knockdown concentration
LC_50_: 50% lethal concentration
RR: resistance ratio
*vgsc*: voltage gated sodium channel
CDC: Centers for Disease Control and Prevention
WHO: World Health Organization
DDT: dichlorodiphenyltrichloroethane
PCR: polymerase chain reaction
MFOs: mixed-function oxidases
GSTs: glutathione S-transferases
